# Metagenomic insights into the microbe-mediated B and K_2_ vitamin biosynthesis in the gastrointestinal microbiome of ruminants

**DOI:** 10.1186/s40168-022-01298-9

**Published:** 2022-07-21

**Authors:** Qian Jiang, Limei Lin, Fei Xie, Wei Jin, Weiyun Zhu, Min Wang, Qiang Qiu, Zhipeng Li, Junhua Liu, Shengyong Mao

**Affiliations:** 1 Centre for Ruminant Nutrition and Feed Technology Research, College of Animal Science and Technology, Nanjing Agricultural University, Nanjing, China; 2 Laboratory of Gastrointestinal Microbiology, Jiangsu Key Laboratory of Gastrointestinal Nutrition and Animal Health, College of Animal Science and Technology, Nanjing Agricultural University, Nanjing, China; 3 National Center for International Research on Animal Gut Nutrition, Nanjing Agricultural University, Nanjing, China; 4 CAS Key Laboratory for Agro-Ecological Processes in Subtropical Region, Institute of Subtropical Agriculture, Chinese Academy of Sciences, Changsha, China; 5 School of Ecology and Environment, Northwestern Polytechnical University, Xi’an, China; 6 College of Animal Science and Technology, Jilin Agricultural University, Changchun, China

**Keywords:** B and K_2_ vitamins, Ruminants, Gastrointestinal microbiome, Microbial genomes, Cobalamin, High-grain diet

## Abstract

**Background:**

B and K_2_ vitamins, essential nutrients in host metabolism, can be synthesized by the rumen microbiome in ruminants and subsequently absorbed by the host. However, the B and K_2_ vitamin biosynthesis by the whole gastrointestinal microbiome and their abundances in different dietary strategies are largely unknown. Here, we reanalyzed our previous large-scale metagenomic data on the gastrointestinal microbiome of seven ruminant species and recruited 17,425 nonredundant microbial genomes from published datasets to gain a comprehensive understanding of the microbe-mediated B and K_2_ vitamin biosynthesis in ruminants.

**Results:**

We identified 1,135,807 genes and 167 enzymes involved in B and K_2_ vitamin biosynthesis. Our results indicated that the total abundances of B and K_2_ vitamin biosynthesis were dominant in the stomach microbiome, while the biosynthesis of thiamine, niacin, and pyridoxine was more abundant in the large intestine. By examining 17,425 nonredundant genomes, we identified 2366 high-quality genomes that were predicted to de novo biosynthesize at least one vitamin. Genomic analysis suggested that only 2.7% of these genomes can synthesize five or more vitamins, and nearly half of genomes can synthesize only one vitamin. Moreover, we found that most genomes possessed cobalamin transporters or cobalamin-dependent enzymes to consume cobalamin directly, and only a few microbial genomes possessed a complete cobalamin biosynthesis pathway. Based on these genomic data, we examined the effect of the high-grain (HG) diet on the vitamin biosynthesis of the rumen microbiome of dairy cattle. We revealed that most vitamin biosynthesis was enhanced in the HG group, while only cobalamin synthesis was inhibited in the HG group, indicating that dietary fiber is vital for cobalamin biosynthesis.

**Conclusions:**

We primarily provided a gene catalog and 2366 microbial genomes involved in B and K_2_ vitamin biosynthesis in ruminants. Our findings demonstrated the regional heterogeneity and dietary effect of vitamin biosynthetic potential in the ruminant gastrointestinal microbiome and interpreted the biosynthesis mechanisms of these microbes and their physiological adaptability. This study expands our understanding of microbe-mediated vitamin biosynthesis in ruminants and may provide novel targets for manipulation to improve the production of these essential vitamins.

Video abstract.

**Supplementary Information:**

The online version contains supplementary material available at 10.1186/s40168-022-01298-9.

## Background

The gastrointestinal tract (GIT) of ruminants is symbiotic with trillions of microorganisms, which in turn supply the animal host with essential nutrients [1, 2], such as volatile fatty acids (VFAs), amino acids from microbial proteins, and vitamins. Of which, vitamins B and K_2_ (menaquinone) are indispensable coenzymes for many metabolic pathways [3] and genetic information processing [4], which are crucial for growth and health in ruminants [5]. For example, vitamin B_12_ is a coenzyme for methylmalonyl-CoA mutase involved in gluconeogenesis, which is further associated with milk production [6]. In addition, the deficiencies of B vitamins and VK_2_ in ruminants may harm animal reproductive and production performance [7–9], such as clinical lameness caused by biotin deficiency [10] and anorexia and polioencephalomalacia caused by thiamine deficiency [11]. Therefore, an exogenous supply of vitamins to meet the needs of animal production has prevailed for decades [12]. Regarding that the supplements are costly, strategies to elevate the vitamin biosynthetic capacities of GIT microbiota and decrease the supply of exogenous vitamins are imperative for reducing animal production costs. Hence, an understanding of B vitamins and VK_2_ biosynthesis via the ruminant GIT microbiome is necessary to develop strategies to modulate the amount of these vitamin biosyntheses in the ruminant GIT.

Vitamin biosynthesis from microorganisms has been found in the human gut and rumen [3, 13, 14]. For instance, thiamine can be biosynthesized via a separate formation of thiazole and pyrimidine. The biosynthesis of biotin has five routes (*bioW* for pimelate and *bioZ*, *bioG*, *bioH*, and *bioK* for malonyl-ACP) from malonyl-ACP or pimelate. Specially, cobalamin has the most complex biosynthesis pathway among B vitamins, which can only be de novo biosynthesized by prokaryotes through two routes: aerobic or anaerobic pathway, according to the time of cobalt insertion and the oxygen requirement [15]. In detail, precorrin 2 was the first precursor committed to these two pathways, and cobalt chelation occurs via *cbiK* in the anaerobic pathway, while via *cobNST* in the aerobic pathway. Studies based on microbial cultivation have pointed out that some specific rumen microbes can synthesize B vitamins, such as *Corynebacterium vitaeruminis* DSM 20294^T^ [16], *Fibrobacter succinogenes* [17], and *Selenomonas ruminantium* [18]. In addition, the relationships between some ruminal microbes (e.g., *Prevotella*, *Bacteroides*, and *Ruminococcus 1*) and B vitamin abundance based on 16S rRNA sequencings are also reported [19, 20].

Unlike humans, ruminants have evolved a compartmentalized stomach and their complete GIT contains 10 distinct physical compartments (rumen, reticulum, omasum, abomasum, duodenum, jejunum, ileum, cecum, colon, and rectum). These spatially specialized regions were uncovered to harbor a dissimilar microbial community, resulting from transformations in physiology substrate availabilities, retention time of digesta, and pH levels [21–24]. Previous studies have recognized that the GIT microbial composition and function exhibit a regional heterogeneity, and the lower GIT microbiota plays a previously overlooked role in ruminant production and health [24–26]. However, previous studies on vitamin biosynthesis in ruminant have mainly focused on the rumen microbiota [12, 27], and the comprehensive understanding across the whole GIT regions is lacking. Therefore, whether B and K_2_ vitamin biosynthesis from microbiome are widespread in the GIT and how the non-rumen regions contributed to the vitamin pool is needed to further explore. In addition, understanding the complex biosynthesis process of B and K_2_ vitamins throughout the GIT is key to informing strategies for the subsequent targeted regulation of gastrointestinal microbiota to improve vitamin production and utilization in ruminants. Furthermore, evidence has been identified that changing the ratio of forage to concentrate in the dietary regime can alter the vitamin concentrations in the rumen of calves, sheep, and steers [28]. However, the contribution of microbe-mediated vitamin biosynthesis and how the shifts in microbial composition are related to the production of vitamins remain unclear.

Here, we identified 1,135,807 genes and 2366 high-quality genomes involved in B and K_2_ vitamin biosynthesis from our previous study [24] and public datasets [29–36]. Using these reference datasets, we demonstrated the regional heterogeneity of the GIT microbiome in B and K_2_ vitamin biosynthesis in ruminants. In addition, our genomic analysis provided deep insights into cobalamin synthesis system present in the GIT microbiome, and a comprehensive understanding of microbe-mediated vitamin biosynthesis in response to a high-grain diet perturbation. The findings may provide novel solutions for developing effective strategies to enhance microbial B and K_2_ vitamin biosynthesis in ruminant GIT.

## Results

### B and K_2_ vitamin biosynthesis abundance throughout the GIT

**Fig. 1:**
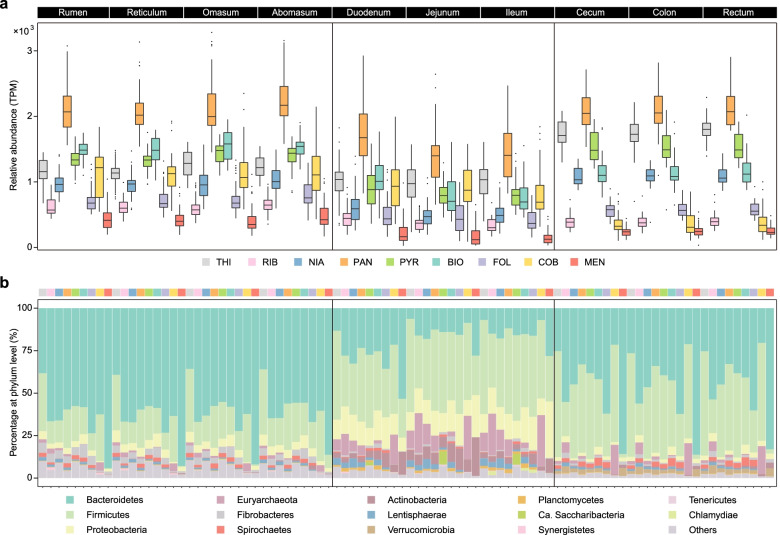
The relative abundance and taxonomic distribution of B and K_2_ vitamin de novo biosynthesis. **a** Total abundance of the microbial genes related to B and K_2_ vitamin biosynthesis throughout the 10 GIT regions. The horizontal lines indicate the medians, and the whiskers indicate the lowest and highest points within 1.5 × the interquartile ranges into the lower and upper quartiles, respectively. **b** Phylogenetic distribution of B and K_2_ vitamin biosynthetic genes at the phylum level among 10 GIT regions. B and K_2_ vitamins are abbreviated as: thiamine (THI), riboflavin (RIB), niacin (NIA), pantothenate (PAN), pyridoxine (PYR), biotin (BIO), folate (FOL), cobalamin (COB), and menaquinone (MEN)

To illustrate the regional variations of microbe-mediated vitamin biosynthesis, we identified 1,135,807 genes and 167 KEGG orthologies (KOs) involved in eight B vitamins (thiamine, riboflavin, niacin, pantothenate, pyridoxine, biotin, folate, and cobalamin) and K_2_ (menaquinone) de novo biosynthesis pathways (Additional file 2: Table S1) based on the published ruminant gastrointestinal microbial gene catalog generated from the 370 GIT samples of seven ruminant species (dairy cattle, water buffalo, yak, goat, sheep, roe deer, and water deer) [24]. We first assessed the abundances of genes involved in B and K_2_ vitamin biosynthesis pathways and found that the pathway abundance involved in pantothenate biosynthesis was the highest among the GIT regions, while menaquinone exhibited the lowest pathway abundance (Fig. 1a; Additional file 1: Fig. S1–S9). We also found that the pathway abundance of B and K_2_ vitamin biosynthesis had a high variance across the whole GIT regions (Fig. 1a), and this was supported by the results of a variance partitioning analysis (VPA), which showed that separated regions accounted for more than half (50.6%) of the explanatory degree of variation in B and K_2_ vitamin biosynthesis (Additional file 1: Fig. S10). For example, the biosynthesis pathways of riboflavin, pantothenate, biotin, folate, cobalamin, and menaquinone were more prevalent in the stomach (rumen, reticulum, omasum, and abomasum), while thiamine, niacin, and pyridoxine were more enriched in the large intestine (cecum, colon, and rectum). These results suggested that vitamin biosynthesis mainly functioned by the microbes in the stomach and large intestine of ruminants. Furthermore, VPA showed that pyridoxine (62.2%) and niacin (59.6%) were the two most affected vitamins by regional separation (Additional file 1: Fig. S10). Indeed, the requirements of pyridoxine and niacin for ruminants are mainly dependent on GIT microbial biosynthesis, due to the low pyridoxine content in forage and the dietary niacin destructed by the rumen environment [37].

To further characterize the biosynthesis process of these vitamins, we examined all the alternative pathways present in the GIT microbiome. For thiamine, its biosynthesis pathway comprises two branches, and the branch with *thiH* was prevalent in the GIT microbiome (Additional file 1: Fig. S1; Additional file 2: Table S2). The *NaMNAT* branch of the niacin biosynthesis pathway was more prevalent in the GIT microbiome compared to the *NMNAT* branch (Additional file 1: Fig. S3; Additional file 2: Table S2). The biosynthesis pathway of pyridoxine had two branches, and most genes in the longer branch showed a higher relative abundance, except for *pdxH*, which restricted the end of the process (Additional file 1: Fig. S5; Additional file 2: Table S2), suggesting that the GIT microbiome may have a low potential to synthesize pyridoxine. For biotin, four biosynthesis branches existed in the ruminant GIT microbiome, but the *bioC*-*bioZ* and *bioW* branches were more prevalent in ruminants (Additional file 1: Fig. S6; Additional file 2: Table S2). Menaquinone was synthesized through the classical pathway (mainly comprising the functional roles named using prefix *men*) or the futalosine pathway. The classical pathway was more enriched in the ruminant GIT microbiome (Additional file 1: Fig. S9; Additional file 2: Table S2). Generally, these results indicate that the alternative pathways of each vitamin biosynthesis are selectively prevalent in the ruminant GIT microbiome and several vitamins are dampened to be synthesized by microbiota, such as pyridoxine.

### Phylogenetic origin of vitamin biosynthetic genes

To gain insights into microbe-mediated vitamin metabolism, we performed a taxonomic analysis of these vitamin biosynthetic genes. Our results showed that the B and K_2_ vitamin biosynthetic genes were phylogenetically assigned to the phyla *Bacteroidetes* (44.24%), followed by *Firmicutes* (33.24%) and *Proteobacteria* (6.15%; Fig. 1b), which differ from those of humans (prevalent by *Firmicutes* and *Proteobacteria*) [14]. Notably, the assigned phylum in the stomach was mainly *Bacteroidetes*, while members of the phylum *Euryarchaeota* were crucial executors of vitamin biosynthesis in the small intestine. In addition, the biosynthetic genes related to riboflavin, niacin, folate, and menaquinone were mainly derived from *Bacteroidetes*, accounting for more than 50%, especially menaquinone (up to 76.37%). For cobalamin, 12.08% of biosynthetic genes were classified into the phylum *Euryarchaeota*, whose proportion was much higher than other vitamins (Additional file 1: Fig. S11; Additional file 2: Table S3).

The most assigned genera for the B and K_2_ vitamin biosynthetic genes were *Prevotella*, *Bacteroides*, *Clostridium*, *Ruminococcus*, *Methanobrevibacter*, *Fibrobacter*, and *Alistipes* (Additional file 1: Fig. S12). The most prevalent taxa for vitamin biosynthesis in the stomach were derived from the genus *Prevotella*, while the predominant genera in the small and large intestine were diverse. In the large intestine, the most predominant genera for vitamin biosynthesis were *Bacteroides* and *Alistipes*, except for thiamine, which was mainly synthesized by *Clostridium*. For the small intestine, *Methanobrevibacter* spp. contributed to the biosynthesis of more than half of the B vitamins, such as riboflavin and cobalamin. Together, the dominant microbiota involved in B and K_2_ vitamin biosynthesis are distinct throughout the GIT regions, and different vitamins have specific microbial populations in the GIT microbiome.

In addition, the Random Forest model was used to identify the important indicator of vitamin biosynthesis across GIT regions, and we indicated that the richness index (mean square error = 18.99%), and the abundance of *Prevotella* spp. (23.33%) and *Bacteroides* spp. (21.53%) were the most important indicators in the varied GIT vitamin biosynthesis (Additional file 1: Fig. S13). These results suggest that vitamin synthesis is closely linked to the colonization of microbes in the GIT, especially these two bacteria. Optimal information flow and convergent cross mapping (CCM) [38, 39] have been described well to detect the casual interactions in ecosystem; thus, we used the CCM model to further explore whether the structure and distribution of the GIT microbial community are related with vitamin biosynthesis. We found that the ratio of *Bacteroidetes* to *Firmicutes* had a strong causal interaction with GIT vitamin biosynthesis (Additional file 1: Fig. S14), suggesting that the nutrient-mediated microbial community structure in GIT regions shaped the vitamin biosynthesis, which is also consistent with the previous study in the human gut [3].

### Cobalamin de novo biosynthesis in ruminant GIT microbiome

**Fig. 2:**
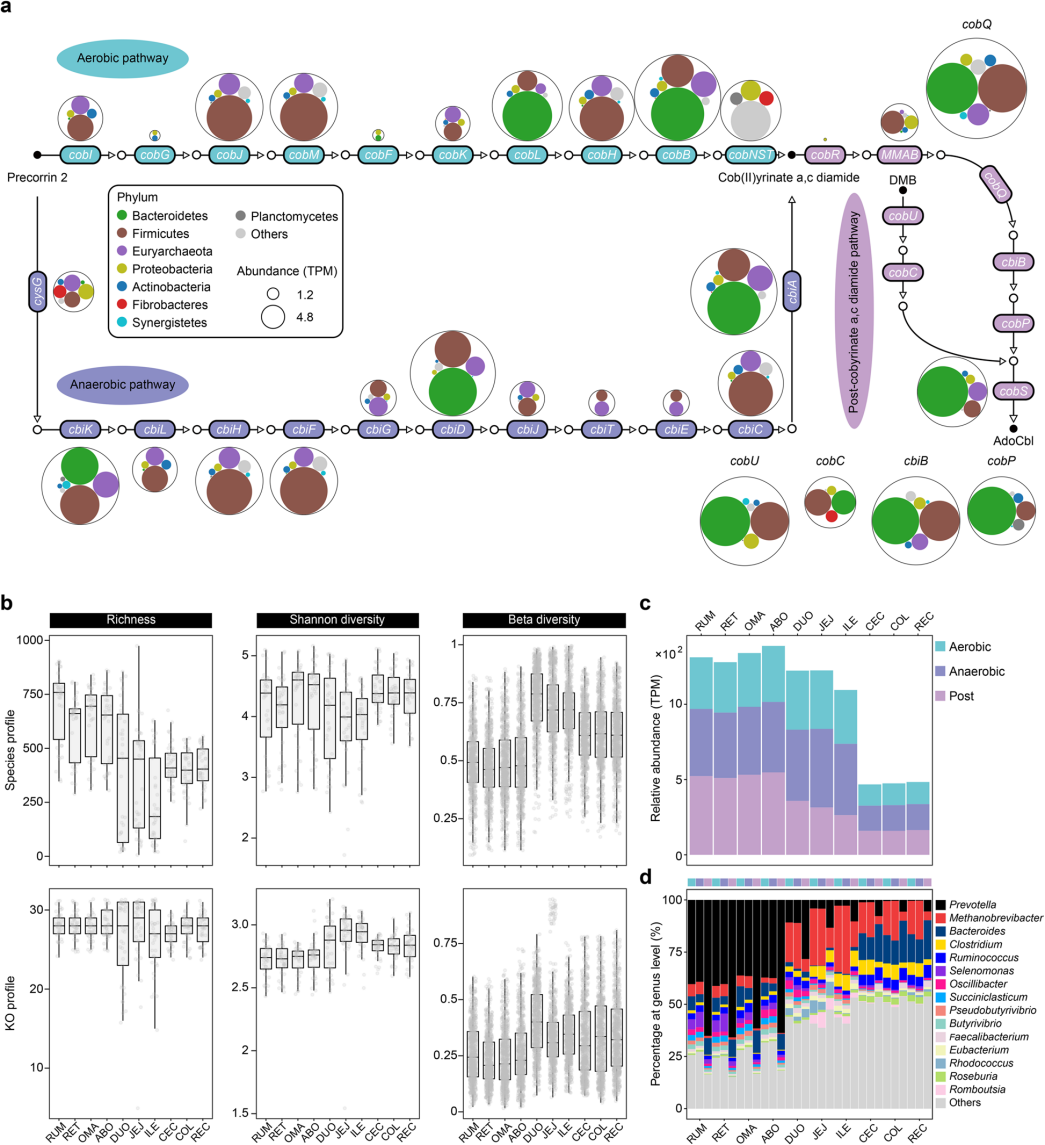
Cobalamin de novo biosynthesis in the ruminant GIT microbiome. **a** The de novo biosynthesis pathway of cobalamin. The circle packing represents the phylogenetic origin of the corresponding functional role at the phylum level, and circle size represents the relative abundance (TPM). Rectangles represent functional roles and circles represent metabolites. **b** The diversity indices of cobalamin biosynthesis based on the species and KO profile among 10 GIT regions. **c** The relative abundances of three parts of the cobalamin biosynthesis pathway among 10 GIT regions. **d** Phylogenetic distribution of the functional roles of three parts of the cobalamin biosynthesis pathway. The GIT regions are abbreviated as rumen (RUM), reticulum (RET), omasum (OMA), abomasum (ABO), duodenum (DUO), jejunum (JEJ), ileum (ILE), cecum (CEC), colon (COL), and rectum (REC)

Cobalamin is an unusual vitamin synthesized exclusively by prokaryotes [40]. The microbe-mediated biosynthesis of cobalamin occurs via two alternative routes according to the timing of cobalt insertion and the requirement of molecular oxygen, including the aerobic and anaerobic pathway (Fig. 2a). In the aerobic pathway, precorrin 2 was methylated into precorrin 3A by *cobI* and finally chelated with cobalt via *cobNST*. By contrast, precorrin 2 was chelated with cobalt to produce cobalt-sirohydrochlorin in the second step of anaerobic pathway. The aerobic and anaerobic pathways converged into cob(II)yrinate a,c diamide, which was finally converted into a cobamide coenzyme (adenosylcobalamin). Here, the abundance of *cobG* and *cobF* was low (TPM < 10) in the aerobic pathway, indicating that this pathway is rarely present in the anaerobic environment of the ruminant GIT (Additional file 2: Table S2). Several genes (e.g., *cbiG*, *cbiJ*, *cbiT*, and *cbiE*) with low abundance in the anaerobic pathway also indicate that the de novo biosynthesis of cobalamin is likely restricted in the ruminant GIT microbiome (Fig. 2a).

When exploring the taxonomic assignment of cobalamin biosynthesis among the GIT regions, we observed that the alpha diversity of taxonomic populations was the highest in the stomach and the lowest in the small intestine, while beta diversity showed the opposite trend (Fig. 2b). We also found that the biosynthetic genes enriched in the stomach (e.g., *cobBCLQUPS* and *cbiBDK*) were mainly classified into the phyla *Bacteroidetes* and *Firmicutes*, while *cobIKHL* and *cbiHFG* enriched in the small intestine were classified into the phyla *Firmicutes* and *Euryarchaeota* (Fig. 2a). We further observed the differential distribution of taxa at the genus level across the GIT regions whether in the aerobic pathway, anaerobic pathway, or post-cobyrinate a,c diamide pathway (Fig. 2d). For example, *Prevotella* was the most assigned genus for most genes related to cobalamin de novo biosynthesis in the stomach, and *Methanobrevibacter* was the major contributor to cobalamin biosynthesis in the intestine, especially in the small intestine. In addition, *Bacteroides*, *Clostridium*, and *Ruminococcus* were also prevalent genera for cobalamin biosynthesis in the large intestine.

### Compendium of 2366 vitamin de novo biosynthetic genomes

**Fig. 3:**
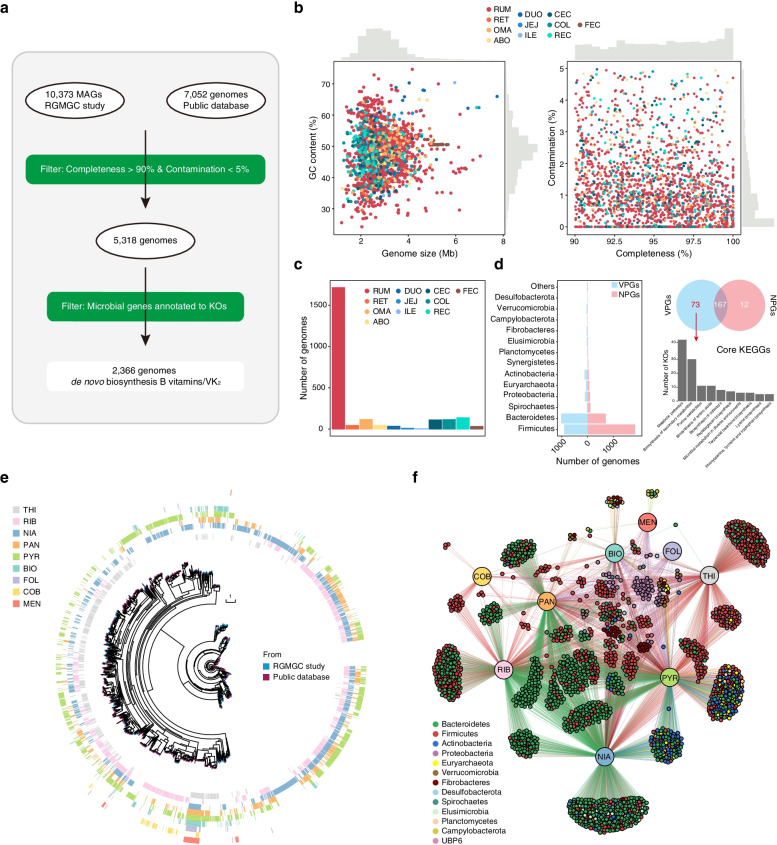
2366 genomes identified to synthesize B and K_2_ vitamins. **a** The workflow of identifying genomes that can synthesize B and K_2_ vitamins. **b** Genomic statistics for 2366 VPGs. **c** The number of genomes retrieved from the GIT regions. **d** Genome comparison of taxonomy and core KOs between VPGs and NPGs. **e** The maximum-likelihood tree of 2366 VPGs. Clades are colored according to the source of genomes. The heatmaps in the outer layer show that the corresponding genome has vitamin synthesis capabilities (colored) or not (blank). **f** Correlation network of vitamins and genomes, and genomes are colored with taxonomic information. The GIT regions are abbreviated as rumen (RUM), reticulum (RET), omasum (OMA), abomasum (ABO), duodenum (DUO), jejunum (JEJ), ileum (ILE), cecum (CEC), colon (COL), rectum (REC), and feces (FEC). B vitamins and VK_2_ are abbreviated as thiamine (THI), riboflavin (RIB), niacin (NIA), pantothenate (PAN), pyridoxine (PYR), biotin (BIO), folate (FOL), cobalamin (COB), and menaquinone (MEN)

To further explore the B and K_2_ vitamin biosynthetic capabilities of the ruminant GIT microbiome at the genome level, we recruited 17,425 nonredundant genomes (10,373 MAGs from our previous study [24] and 7052 genomes from the collection of public ruminant microbial genomes [29–36]; Additional file 3: Table S5). After the quality evaluation using CheckM (https://github.com/Ecogenomics/CheckM/wiki) [41], we obtained 5318 high-quality (> 90% completeness and < 5% contamination) genomes (Fig. 3a). For genomic annotation analysis, 2366 genomes were predicted to de novo synthesize at least one B or K_2_ vitamin (Fig. 3a; Additional file 3: Table S4, S7). These 2366 genomes ranged in size from 1.19 to 7.74 megabases (Mb), with GC content ranging from 24.21 to 74.64% (Fig. 3b; Additional file 3: Table S7). Of these, 1714 genomes were assembled from the rumen, followed by 138, 116, 114, and 111 genomes from the rectum, omasum, colon, and cecum, respectively (Fig. 3c; Additional file 3: Table S7). The taxonomic assignment revealed that 1024 and 905 genomes were assigned to the top two phyla, *Bacteroidetes* (43.3%) and *Firmicutes* (38.3%), respectively, followed by 114 genomes for *Actinobacteria* (4.8%), 106 genomes for *Proteobacteria* (4.5%), and 58 genomes for *Euryarchaeota* (2.5%) (Fig. 3d; Additional file 3: Table S6).

After the assessment of vitamin synthetic capacity in these genomes, we found that 1135 genomes can synthesize one type of vitamin, 1167 genomes can synthesize two to four types of vitamins, and 32 genomes can synthesize seven types of vitamins (Fig. 3e; Additional file 3: Table S7). Notably, we found that no genome could synthesize all nine vitamins de novo, and only four genomes could synthesize eight vitamins, including two genomes assigned to *Firmicutes* (lack of cobalamin biosynthesis) and another two genomes assigned to *Proteobacteria* (lack of riboflavin biosynthesis) (Fig. 3e; Additional file 3: Table S7). This observation suggests that most microbes may obtain their non-synthetic vitamins through interactions between microorganisms. For each vitamin, 544 genomes were classified into thiamine biosynthesis, 895 for riboflavin, 1190 for niacin, 631 for pantothenate, 1017 for pyridoxine, 160 for biotin, 42 for folate, 53 for cobalamin, and 53 for menaquinone (Fig. 3e, f). In addition, 203 thiamine-producing genomes were mainly assigned to the phylum *Firmicutes* (81.3%) and 371 niacin-producing genomes were mainly classified into the phylum *Bacteroidetes* (63.6%) (Fig. 3f). Notably, all genomes from the genera *Salmonella* and *Escherichia* can synthesize seven vitamins, suggesting that the members of *Salmonella* spp. and *Escherichia* spp. have a wide range of vitamin biosynthetic capabilities (Additional file 1: Fig. S15; Additional file 3: Table S7). We also found that folate was mainly synthesized by *Proteobacteria*-affiliated genomes (81.0%), and cobalamin was mainly synthesized by *Firmicutes-*affiliated genomes (86.8%). Interestingly, we found that *Bacteroidetes*-affiliated genomes could not synthesize folate and cobalamin in these 2366 genomes (Fig. 3f). However, most *Bacteroidetes*-affiliated genomes have been predicted to synthesize folate and cobalamin in the human gut [3]. These findings indicate that there might be variations in vitamin biosynthetic abilities between human and ruminant GIT microbiota.

To explore the special features of these 2366 genomes with the capacity to synthesize B and K_2_ vitamins, we compared these vitamin-producing genomes (VPGs) with other non-producing genomes (NPGs) at the taxonomic and functional levels. We observed that several phyla contained more annotated genomes to synthesize vitamins, including *Bacteroidetes*, *Proteobacteria*, *Actinobacteria*, *Elusimicrobia*, *Fibrobacteres*, *Campylobacterota*, *Verrucomicrobia*, and *Desulfobacterota* (Fig. 3d). The ratio of *Bacteroidetes* to *Firmicutes* in VPGs is much higher than in NPGs, which was in accordance with the ration of *Bacteroidetes* to *Firmicutes* having a strong casual interaction with vitamin biosynthesis. To further explore the unique functions of 2366 VPGs, we compared the metabolic pathways between VPGs and NPGs. Thus, we obtained 73 core KOs only present in VPGs, associated with several functional metabolism pathways, including biosynthesis of secondary metabolites, purine metabolism, biosynthesis of amino acids, biosynthesis of cofactors, and peptidoglycan biosynthesis (Fig. 3d; Additional file 3: Table S8). These results suggest that microorganisms use vitamins for primary and secondary metabolisms, such as carbon catabolism, amino acid biosynthesis, nucleotide biosynthesis, and natural product biosynthesis [42]. Thus, vitamins could be essential coenzymes for maintaining secondary metabolite biosynthesis, purine metabolism, and amino acid biosynthesis in the ruminant GIT microbiome [43].

### Comparative genomics of the 675 genomes related to cobalamin biosynthesis

**Fig. 4:**
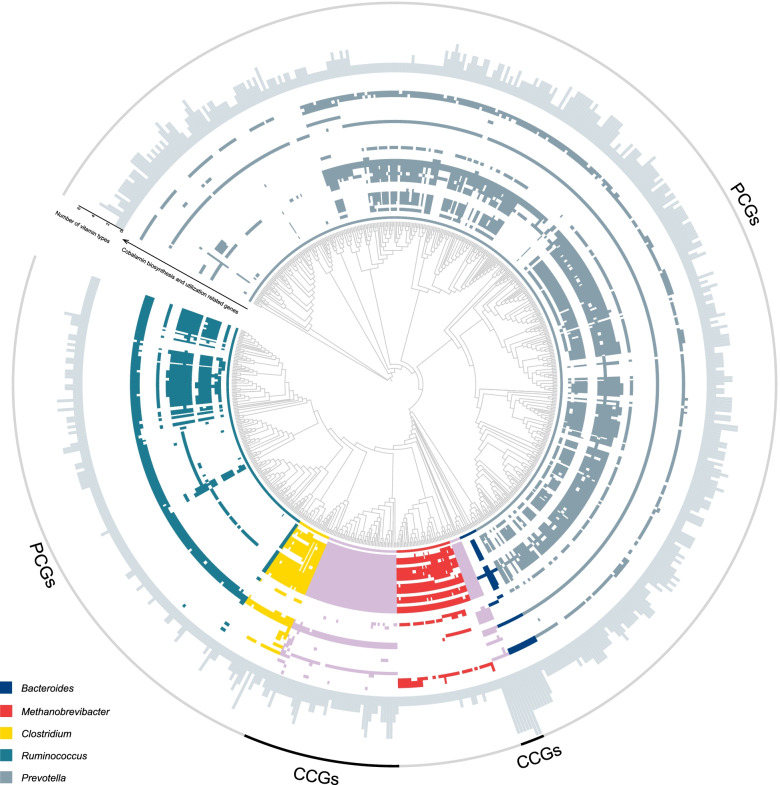
Genomic comparison of cobalamin biosynthesis of 675 VPGs. The maximum likelihood tree was constructed using PhyloPhlAn [44]. The heatmaps show the presence (colored) or absence (white) of dispensable functional roles for cobalamin de novo biosynthesis or cobalamin transporters and cobalamin-dependent enzymes in the corresponding genomes. Bar graphs in the outer layer represent the number of vitamin types synthesized by the corresponding genomes. The genes related to cobalamin biosynthesis and utilization, including *cysG*, *cbiK*, *cbiL*, *cbiH*, *cbiG*, *cbiF*, *cbiD*, *cbiJ*, *cbiT*, *cbiE*, *cbiC*, *cbiA*, *MMAB*, *cobQ*, *cbiB*, *cobP*, *cobS*, *cobI*, *cobG*, *cobF*, *cobL*, *cobNST*, *btuB*, *btuF*, *btuC*, *btuD*, *cbrT*, *cbrV*, *metH*, *pduCDE*, *dhaB*, *mal*, *eutBC*, *kamDE*, *ordSE*, *glmES*, *MCM*, *nrdJ*, *mcr*, *mtaA*, *mtaB*, *mtmB*, and *mttB*, are represented by the inner circle to the outer circle, respectively

The cobalamin de novo biosynthesis pathway was mainly contributed by the members of the genera *Prevotella*, *Methanobrevibacter*, *Bacteroides*, *Clostridium*, and *Ruminococcus* (Fig. 2d). However, genomes affiliated with these five genera contained incomplete genes for de novo cobalamin biosynthesis (Fig. 4). Thus, the question arises about which genes are missing in these genomes that restrict them from synthesizing cobalamin de novo. To address this, we compared 622 genomes (PCGs) belonged to these five genera that contained part of genes involved in cobalamin biosynthesis with 53 genomes (CCGs) that possessed complete cobalamin de novo biosynthesis genes. We found that the CCGs contained all indispensable genes in the anaerobic pathway and post-cobyrinate a,c diamide pathway but lacked the most indispensable genes in the aerobic pathway, suggesting that they processed the cobalamin synthesis via the anaerobic pathway (Additional file 4: Table S9). Conversely, the PCGs lacked the most indispensable genes (*cobI*, *cobG*, *cobF*, and *cobNST*) in the aerobic pathway, which were involved in corrin ring synthesis (convert precorrin 2 to precorrin 6A) and cobalt chelation (Additional file 4: Table S9; Additional file 2: Table S1). Moreover, most *Prevotella*-affiliated genomes (> 90%) lost five indispensable genes (i.e., *cysG*, *cbiL*, *cbiJ*, *cbiT*, and *cbiE*) in the anaerobic pathway; *Bacteroides*-affiliated genomes lacked *cysG*, *cbiT*, and *cbiE* genes; *Methanobrevibacter*- and *Clostridium*-affiliated genomes lacked *cysG*, *cbiH*, *cbiT*, and *cbiE* genes; and *Ruminococcus*-affiliated genomes lacked *cysG*, *cbiH*, and *cbiE* genes. We also found that all *Methanobrevibacter*-affiliated genomes lacked *MMAB* and *cobP* in the post-cobyrinate a,c diamide pathway. The key genes of these PCGs had appropriate complementarity, suggesting that they were expected to synthesize cobalamin collaboratively [45].

We additionally analyzed cobalamin utilization by predicting the presence or absence of genes related to cobalamin transporters and cobalamin-dependent enzymes in the two genomic datasets (Additional file 4: Table S10). This result showed that genes encoded cobalamin transporters were present in both PCGs and CCGs, except for *Prevotella-* and *Bacteroides*-affiliated genomes (Fig. 4; Additional file 4: Table S9). The gene families of the transporter (*cbrV* and *cbrT*) were commonly present in the genomes classified into the class Negativicutes and the genera *Clostridium* and *Ruminococcus* (Additional file 4: Table S9). We also observed that 98.1% of the total genomes in the two genomic datasets contained at least one cobalamin-dependent enzyme, and 99.1% of the genomes had at least one cobalamin transporter or cobalamin-dependent enzyme (Fig. 4; Additional file 4: Table S9). Furthermore, most cobalamin-dependent enzymes were widespread across different metabolism pathways, such as methionine synthase (*metH*), methylmalonyl-CoA mutase (*MCM*), and ribonucleotide reductase (*nrdJ*). These results indicate that the capability of using cobalamin is widespread in ruminant GIT microbiomes, but only a few microbes have a complete cobalamin de novo biosynthesis pathway.

### Effects of the high-grain diet on vitamin biosynthesis of the rumen microbiome

**Fig. 5:**
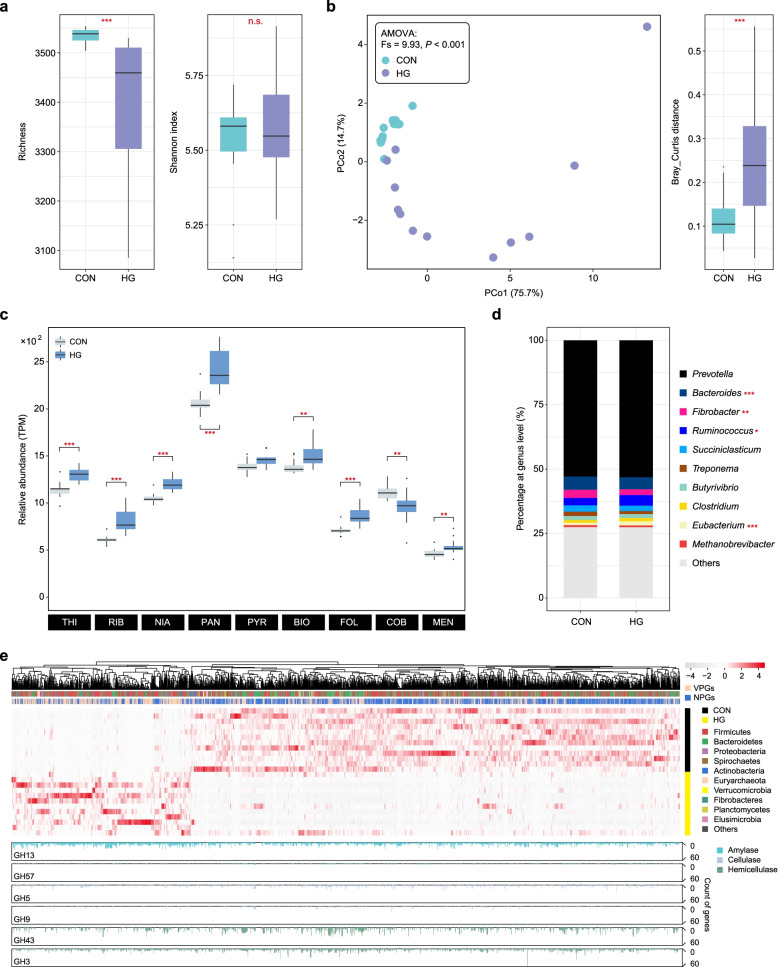
Comparisons of B and K_2_ vitamin biosynthesis between the CON and HG groups. **a** Effects of the HG diet on the alpha diversity of B and K_2_ vitamins at the species level (Wilcoxon rank-sum test, n.s. *P* > 0.05, ***P < 0.001). **b** PCoA of the microbiota related to B and K_2_ vitamin biosynthesis in the two groups. AMOVA analysis showed the significances between the two groups (*P* < 0.001). Bray-Curtis distances between the two groups are shown in the box plot (Wilcoxon rank-sum test, ***P < 0.001). **c** Comparisons of the relative abundance of B and K_2_ vitamin biosynthesis between the two groups (Wilcoxon rank-sum test, **P < 0.01, ***P < 0.001). **d** The relative abundance of the microbiota related to cobalamin biosynthesis between the two groups at the genus level (Wilcoxon rank-sum test, *P < 0.05, ** *P* < 0.01, ***P < 0.001). **e** Differential enrichment of VPGs and NPGs between the two groups. Heatmaps show the relative abundance (TPM) of differentially enriched genomes, and the colored bar represents the relative abundance corrected using the method of *Z*-score. Bar plots represent the gene counts of GH families. B vitamins and VK_2_ are abbreviated as thiamine (THI), riboflavin (RIB), niacin (NIA), pantothenate (PAN), pyridoxine (PYR), biotin (BIO), folate (FOL), cobalamin (COB), and menaquinone (MEN)

The rumen microbiota were the main participants in the GIT vitamin biosynthesis, but it remains unknown how robust the state compositions and functions of rumen vitamin-related microbiota are regarding dietary changes and whether they could be manipulated using dietary interventions [46, 47]. Therefore, we reanalyzed the data from the previous ruminal microbiome in dairy cattle fed with the high-forage (CON) and high-grain (HG) diets using our genomic dataset related to vitamins biosynthesis [48]. We found that the HG diet caused pronounced effects on the B and K_2_ vitamin synthesis microbiome in the rumen (AMOVA, *P* < 0.001; Fig. 5b), underpinned by VPA analysis that dietary shifts accounted for 48.3% of the effects affecting B and K_2_ vitamin biosynthesis (Additional file 1: Fig. S16). We observed significant opposing shifts in alpha and beta diversity of B and K_2_ vitamin biosynthesis-related microbiota, wherein alpha diversity was decreased, and beta diversity was increased in the HG group compared to the CON group (Fig. 5a, b). Genes involved in most vitamin synthesis (i.e., thiamine, riboflavin, niacin, pantothenate, biotin, folate, and menaquinone) were enhanced in the HG group, while only cobalamin synthesis was inhibited in the HG group (Wilcoxon rank-sum test, *P* < 0.01; Fig. 5c), suggesting that cobalamin may need to be supplied to maintain animal health during HG diet feeding. We then performed taxonomic profiling of the cobalamin biosyntheti genes to mine the specific species. After HG diet feeding, significant shifts were detected in the taxa from four genera, namely *Bacteroides*, *Fibrobacter*, *Ruminococcus*, and *Eubacterium* (Wilcoxon rank-sum test, *P* < 0.05; Fig. 5d). Among them, we speculated that the decreased abundances of *Bacteroides* spp. and *Fibrobacter* spp. in the HG group may cause the decrease in cobalamin biosynthesis through the CCM analysis (Additional file 1: Fig. S17). In addition, Random Forest analysis was used to explore the important taxonomic and metabolic indicators during the dietary change, and we found that the taxa assigned to *Bacteroides* spp. (mean square error = 11.66%), and the biosynthesis pathway abundance of three vitamins, including riboflavin (9.87%), niacin (9.24%), and thiamine (7.07%), were the most significant variables during the diet shifts (Additional file 1: Fig. S18).

We further examined the changes in microbial genomes during dietary perturbation and then found that the abundances of 2520 high-quality genomes were significantly altered (Wilcoxon rank-sum test, *P* < 0.05). Among the genomes, 1849 (73.4%) were enriched in the CON group, and 671 (26.6%) were enriched in the HG group (Fig. 5e; Additional file 5: Table S11). Among these differentially enriched genomes, genomes involved in the de novo biosynthesis of B and K_2_ vitamins accounted for 31.0% in the CON group and 63.5% in the HG group, respectively. Furthermore, the HG-enriched genomes were mainly assigned to the phylum *Firmicutes* (230, 54.0%), while the CON-enriched genomes were mainly classified into the phylum *Bacteroidetes* (288, 50.2%). At the functional level, the starch-degrading enzymes (e.g., GH13 and GH77) were significantly predominant in the HG-enriched genomes, which were mainly affiliated with the genera *Sharpea*, *Agathobacter*, *Lachnospira*, and *Lachnobacterium* (Additional file 5: Table S11). In contrast, *Fibrobacter*-affiliated genomes were enriched in the CON group with the prevalence of enzymes involved in fiber-degrading (e.g., GH9, GH51, GH74, GH3, and GH43) (Fig. 5e; Additional file 5: Table S11, S12). These results may explain why most vitamin biosynthesis was elevated with increased starch in the rumen after HG diet feeding. In addition, the decreased fiber-degrading *Fibrobacter* spp. further deciphered the reduction of cobalamin biosynthesis after feeding the HG diet.

## Discussion

Vitamins, especially B and K_2_, can be synthesized by microbiota and mediate fundamental biological processes in microbes and host cells, representing an attractive target for impacting diverse host-microbe symbioses. Unfortunately, till now, the biosynthesis of B and K_2_ vitamins in the ruminant GIT microbiome have been largely unknown, impeding efforts to manipulate ruminant host-microbe symbioses toward stability and health.

Here, we used previous large-scale metagenomic data on the gastrointestinal microbiome of ruminants and recruited 17,425 nonredundant microbial genomes from published datasets to gain a comprehensive understanding of the microbe-mediated vitamin biosynthesis in the ruminant GIT microbiome. Through this vitamin-related microbial gene catalog, we demonstrated the regional heterogeneity of B and K_2_ vitamin biosynthetic abundance and related microbiota from the proximal to distal GIT microbiome of ruminants. The pathway abundances of riboflavin, pantothenate, biotin, folate, cobalamin, and menaquinone biosynthesis were more prevalent in the stomach, while the pathway abundances of thiamine, niacin, and pyridoxine biosynthesis were more enriched in the large intestine. Mounting evidences demonstrated that the large intestine can absorb several microbe-mediated water-soluble vitamins via a specialized transport system [49], and these absorbed vitamins can serve as nutrients for the host and regulate the immunity system [2]. Therefore, the hindgut microbiota also contributes to the vitamin pool in the GIT microbiome of ruminants, which assists in maintaining animal homeostasis.

Furthermore, we identified 2366 genomes that were predicted to de novo synthesize at least one B or K_2_ vitamin. However, only 2.7% of these VPGs can synthesize five or more vitamins, and most genomes can only synthesize one vitamin. These results indicate that most microbes in the GIT are auxotrophs, which may obtain the vitamins from their neighboring vitamin producers via symbiotic relationships, and such cross-feeding stabilizes of the microbial communities [50, 51]. By predicting the core KOs of these 2366 VPGs, we indicated that the imperative trophic metabolisms to microbial communities and host animals cooperated with vitamin biosynthesis. These metabolism pathways contribute greatly to microbial survival, including secondary metabolites as antimicrobial compounds to support their colonization [52], nucleotides as substrates for DNA and RNA synthesis to regulate cellular processes [53], and amino acids as precursors for energy source [54]. Together, vitamin-producing microbes play an indispensable role in the GIT microbial community and host health.

Cobalamin, an unusual vitamin, is exclusively synthesized by a small set of bacteria and archaea [42, 55]. Most of the cobalamin producers in our study were assigned to members of the class Negativicutes (Additional file 4: Table S7), which is consistent with a previous study on cultured rumen microbiota [29]. By depicting the two alternative routes about the aerobic and anaerobic pathways, we have not found that microbes synthesize cobalamin via aerobic pathway across the GIT regions despite the presence of a small number of aerobic organisms in the digestive tract, such as in the small intestine and adjacent epithelial mucosa, which was also found in the human gut [3]. Moreover, only 2.24% of the microbial genomes can biosynthesize cobalamin de novo, and most genomes lacking full synthetic genes can utilize cobalamin for microbial metabolic processes via cobalamin transporters or cobalamin-dependent enzymes. Therefore, cobalamin is likely a good nutrient model for exploring microbial interactions in the GIT microbiome.

Regarding the compositions and functions of rumen vitamin-related microbiota in response to dietary changes, we found that the vitamin synthetic capacities in the rumen changed considerably with the increase in concentrate contents in the diet. Notably, only genes involved in cobalamin biosynthesis were reduced with HG diet feeding, which is also supported by previous studies on dairy cows [56]. We found that, in the perturbations of taxonomic composition, *Bacteroides* spp. and *Fibrobacter* spp. were major mediators contributing to reducing cobalamin biosynthesis during HG diet feeding. The possible reason was demonstrated by the broad ability to digest fiber polysaccharides of the genera *Bacteroides* [57] and *Fibrobacter* [58]. This finding indicates that the dietary composition affects cobalamin biosynthesis, and dietary fiber may favor cobalamin biosynthesis. These results also suggest that cobalamin supplementation should be appropriate when the dietary fiber content is insufficient during the feeding production of ruminants.

## Conclusions

Here, we used previous large-scale metagenomic data on the gastrointestinal microbiome of ruminants and recruited 17,425 nonredundant microbial genomes from published datasets to gain a comprehensive understanding of the microbe-mediated B and K_2_ vitamin biosynthesis in the ruminant GIT microbiome. We uncovered that B and K_2_ vitamin biosynthesis were mainly performed by microbes in the stomach and large intestine of ruminants that were predominantly distributed in the phyla of *Bacteroidetes*, *Firmicutes*, and *Proteobacteria*, suggesting that hindgut microbiota do contribute to the vitamin pool in the GIT microbiome. Through this GIT vitamin-producing microbial gene catalog, 2366 genomes assigned to multi-phyla that were predicted to de novo synthesize at least one B or K_2_ vitamin were identified, wherein several imperative trophic metabolisms collaborated with vitamin biosynthesis. However, only 2.7% of the VPGs can synthesize five or more vitamins, and nearly half of the genomes can synthesize only one vitamin, indicating that most microbes in the GIT microbiome of ruminants are auxotrophs. Notably, we found that only a few microbial genomes possessed the complete de novo biosynthesis pathway of cobalamin. Yet, most genomes can utilize cobalamin via cobalamin transporters or cobalamin-dependent enzymes. Moreover, only cobalamin biosynthesis was reduced in the rumen microbiome of dairy cattle fed with the HG diet, suggesting that dietary fiber is vital for cobalamin biosynthesis. Therefore, our findings provide novel insights into regulating specific microflora, genes, and diet strategies for manipulation to improve the production of these important vitamins in ruminants.

## Methods

### Metagenomic data collection

Metagenomic data were collected from two previous studies [24, 48], available under accession numbers PRJNA657455 and PRJNA639405. From PRJNA657455, 370 digesta samples were collected from the GIT (stomach: rumen, reticulum, omasum, and abomasum; small intestine: duodenum, ileum, and jejunum; large intestine: cecum, colon, and rectum) of seven ruminant species (dairy cattle, water buffalo, yak, goat, sheep, roe deer, and water deer). From PRJNA639405, 24 digesta samples were collected from the rumen of dairy cattle fed with the CON and HG diets.

### Taxonomic and functional analysis of the vitamin-related microbial gene catalog

We recruited a nonredundant ruminant GIT microbial gene catalog (RGMGC) with 154,335,274 genes from our previous study [24]. Taxonomic assignment and functional annotation were performed using DIAMOND [59] (v.0.9.22) with standard protein Basic Local Alignment Search Tool (BLASTP) searches against the NCBI nonredundant (NCBI-NR, October 2018, https://www.ncbi.nlm.nih.gov/refseq/about/nonredundantproteins/) and Kyoto Encyclopedia of Genes and Genomes (KEGG, v.90.0, https://www.genome.jp/kegg/) [60] databases. Burrows-Wheeler Aligner (BWA-MEM, v.0.7.17, http://bio-bwa.sourceforge.net) [61] with maximal exact matches was used to map high-quality reads of the 370 digesta samples back to the RGMGC, and gene profiles were calculated in transcripts per million (TPM) [62]. The relative abundances of annotated taxa and KOs were calculated according to the gene abundance. Briefly, for the taxonomic (phylum and genus) profiles, the abundances of genes assigned to the same phylum or genus were summed as the abundance of each phylum or genus. The KO profiles were calculated using the same method. We then defined KOs that perform the same function in the vitamin biosynthesis pathway as a functional role (Additional file 2: Table S1) and summed the abundances of KOs as the abundance of the corresponding functional role. The pathway abundance related to B and K_2_ vitamin biosynthesis was calculated as the summation of the abundance of KOs in this pathway. Alpha (richness and Shannon index) and beta (Bray-Curtis distance) diversity were calculated using the R vegan package [63] (v.2.5-6).

### Microbial genome collection and quality evaluation

We collected 10,373 MAGs from our previous study [24] and 7052 genomes from the collection of ruminant microbial genomes [29–36] (for more details, see Additional file 3: Table S5). CheckM [41] (v.1.0.7, https://github.com/Ecogenomics/CheckM/wiki) was used to evaluate the completeness and contamination of all 17,425 genomes based on lineage_wf workflow, and 5318 high-quality (completeness > 90% and contamination < 5%) genomes were retained for the downstream analysis. Genome size was corrected using genomic completeness and contamination according to a previous study [64].

### Phylogenetic, taxonomic, and functional analyses of the 5318 genomes

Prodigal [65] (v.2.6.3) was performed to predict open reading frames (ORFs) in these 5318 high-quality genomes with the parameter “-p single.” Taxonomic classifications of these genomes were assigned using GTDB-Tk [66] (v.0.1.6) based on the Genome Taxonomy Database [67] (https://gtdb.ecogenomic.org/), and functional annotations were performed using DIAMOND [59] (v.0.9.22) with BLASTP searches against the KEGG [60] database (v.90.0). We determined a set of indispensable functional roles referring to the previous study [3] (Additional file 3: Table S4), which must be present in a genome considered a vitamin producer that can synthesize B and K_2_ vitamins de novo. Based on the indispensable functional roles (102), we identified 2366 genomes that were predicted to synthesize B and K_2_ vitamins de novo. Phylogenetic trees were generated based on the concatenated protein sequences using PhyloPhlAn [44] (v.1.0) and subsequently visualized using Evolview [68] (v.3) and iTOL [69] (v.4.3.1). The correlation network was constructed using Gephi [70] (v.0.9.2) based on the biosynthesis abilities related to B and K_2_ vitamins in each genome. KOs present in more than 90% of individuals in each genomic dataset were identified as core functions among VPGs or NPGs.

### Genomic analysis of cobalamin biosynthesis and utilization

We performed a comparative genomic analysis between 53 CCGs and 622 PCGs that are members of the genera *Prevotella*, *Methanobrevibacter*, *Bacteroides*, *Clostridium*, and *Ruminococcus*. KOs assigned to cobalamin transporters (*btuB*, *btuF*, *btuC*, *btuD*, *cbrT*, *cbrV*) and cobalamin-dependent enzymes (*metH*, *pduCDE*, *dhaB*, *mal*, *eutBC*, *kamDE*, *ordSE*, *glmES*, *MCM*, *nrdJ*, *mcr*, *mtaA*, *mtaB*, *mtmB*, *mttB*) (Additional file 4: Table S10) were identified using BLASTP searches against the KEGG [60] database (v.90.0).

### Analysis of the effects of the HG diet on the rumen microbiome of dairy cattle

We reanalyzed the metagenomic data from Mu et al. [48] regarding the rumen microbiome of dairy cattle fed with the CON and HG diets. According to the vitamin biosynthesis functional roles, we identified the microbiota related to B and K_2_ vitamin biosynthesis in the rumen. Alpha (Richness and Shannon index) and beta (Bray-Curtis distance) diversity were calculated using the R vegan package [63] (v.2.5-6). PCoA was performed to show the differences in the microbiota related to B and K_2_ vitamin biosynthesis between the CON and HG groups, and AMOVA was calculated using mothur [71] (v.1.44.2) to assess the significance between the two groups. Then, we used 5318 genomes (including 2366 VPGs) as a genomic database to assign metagenomic samples from the CON and HG groups using BWA-MEM [61]. The abundance of genomes was calculated using TPM, and the Wilcoxon rank-sum test was used for differential analysis between the two groups (*P* < 0.05). We obtained 2520 differentially enriched genomes in the HG and CON groups. Protein sequences encoded by each genome were aligned to the CAZy database [72] (v.7) using HMMER [73] (v.3.2.1). Linear discriminant analysis (LDA) was used to analyze the differences in the GH families, and significant differences were defined as |LDA score| > 3.

### Statistical analysis

The abundance of vitamin biosynthesis pathways, VPGs, and the diversity of vitamin biosynthesis-related taxa were compared using Wilcoxon-rank sum test in R (https://www.r-project.org) [74] with a significance threshold of *P* value < 0.05. The PCoA was compared using AMOVA in mothur [71] (v.1.44.2), and the significance was defined as *P* value < 0.05. Convergent cross-mapping was used to detect causal interactions using the R multispatialCCM package (v.1.0) [75]. Random Forest analysis was used to identify important indicators for regional and dietary heterogeneity using the R rfPermute package (v.2.5.1) [76]. Variance partitioning analysis (VPA) was used to assess the variance between vitamin biosynthesis pathway and related microbiota explained by regions and diets in the R vegan package (v.2.5-6) [63]. Linear discriminant analysis (LDA) was used to analyze the differences in the GH families, and the significance was defined as |LDA score| > 3 and *P* value < 0.05.

## Supplementary Information

**Additional file 1: Fig. S1.** The biosynthesis pathway of thiamine. **Fig. S2.** The biosynthesis pathway of riboflavin. **Fig. S3.** The biosynthesis pathway of niacin. **Fig. S4.** The biosynthesis pathway of pantothenate. **Fig. S5.** The biosynthesis pathway of pyridoxine. **Fig. S6.** The biosynthesis pathway of biotin. **Fig. S7.** The biosynthesis pathway of folate. **Fig. S8.** The biosynthesis pathway of cobalamin. **Fig. S9.** The biosynthesis pathway of menaquinone. **Fig. S10.** Variability in differences of vitamin biosynthesis explained by regions and species. **Fig. S11.** Chord plot of the distribution of vitamin biosynthetic genes among different phyla. **Fig. S12.** Phylogenetic distribution of vitamin biosynthetic genes at the genus level throughout the GIT regions. **Fig. S13.** The important indicators for regional heterogeneity identified by Random Forest model. **Fig. S14.** The detection of causal interactions between structure of GIT microbiota and vitamin biosynthesis pathway. **Fig. S15.** Vitamin synthesis capabilities of 31 genomes assigned to the genera *Salmonella* and *Escherichia*. **Fig. S16.** Variability in differences of vitamin biosynthesis explained by diets. **Fig. S17.** The detection of causal interactions between *Bacteroides* and *Fibrobacter* and cobalamin biosynthesis. **Fig. S18.** The important indicators for dietary shifts identified by Random Forest model.

**Additional file 2: Table S1.** Detailed information for B and K_2_ vitamin biosynthesis. **Table S2.** Relative abundance of 123 functional roles in the B and K_2_ vitamin biosynthesis pathways. **Table S3.** Distribution of vitamin biosynthesis among different phyla.

**Additional file 3: Table S4.** Combination of indispensable functional roles for B and K_2_ vitamin *de novo* biosynthesis. **Table S5.** Collection of 17,425 ruminant microbial genomes. **Table S6.** Taxonomic classification of 5318 high-quality genomes. **Table S7.** Genomic statistics and vitamin synthesis capabilities of 2366 VPGs. **Table S8.** Comparison of KOs between VPGs and other NPGs.

**Additional file 4: Table S9.** Detailed information for cobalamin biosynthesis and utilization of the 675 genomes in Fig. 4. **Table S10.** KOs assigned to cobalamin transporters and cobalamin-dependent enzymes.

**Additional file 5: Table S11.** Taxonomic classification and GH family counts of the 2520 differentially enriched genomes in the CON and HG groups. **Table S12.** Differentially enriched GH families in the CON and HG groups.

## Data Availability

The metagenomic datasets analyzed in this study are available under accession numbers PRJNA657455 and PRJNA639405, which were published in our previous study [24, 48]. The ruminant microbial genomes used in this study are obtained from accession number PRJNA657473 and other studies described in the additional file 3.

## Abbreviations

ABO: Abomasum
AMOVA: Analysis of molecular variance
BIO: Biotin
CCGs: Genomes contained complete cobalamin biosynthetic genes
CCM: Convergent cross mapping
CEC: Cecum
COB: Cobalamin
COL: Colon
DUO: Duodenum
FEC: Feces
FOL: Folate
GH: Glycoside hydrolase
ILE: Ileum
JEJ: Jejunum
KEGG: Kyoto Encyclopedia of Genes and Genomes
KO: KEGG orthology
LDA: Linear discriminant analysis
MAG: Metagenome-assembled genome
MEN: Menaquinone
NIA: Niacin
NPG: Non-producing genome
OMA: Omasum
ORF: Open reading frame
PAN: Pantothenate
PCoA: Principal coordinate analysis
PCGs: Genomes contained part of cobalamin biosynthetic genes
PYR: Pyridoxine
REC: Rectum
RET: Reticulum
RIB: Riboflavin
RUM: Rumen
THI: Thiamine
TPM: Transcripts per million
VFA: Volatile fatty acid
VK_2_: Vitamin K_2_
VPA: Variance partitioning analysis
VPG: Vitamin-producing genome
