# Supplementary material for: Metagenomic insights into the microbe-mediated B and K2 vitamin biosynthesis in the gastrointestinal microbiome of ruminants
Source: Microbiome. 2022 Jul 21;10:109. doi: 10.1186/s40168-022-01298-9 (PMC9306216; doi:10.1186/s40168-022-01298-9)
Supplement: Supplementary file 2 — Additional file 1: Fig. S1. The biosynthesis pathway of thiamine. Fig. S2. The biosynthesis pathway of riboflavin. Fig. S3. The biosynthesis pathway of niacin. Fig. S4. The biosynthesis pathway of pantothenate. Fig. S5. The biosynthesis pathway of pyridoxine. Fig. S6. The biosynthesis pathway of biotin. Fig. S7. The biosynthesis pathway of folate. Fig. S8. The biosynthesis pathway of cobalamin. Fig. S9. The biosynthesis pathway of menaquinone. Fig. S10. Variability in differences of vitamin biosynthesis explained by regions and species. Fig. S11. Chord plot of the distribution of vitamin biosynthetic genes among different phyla. Fig. S12. Phylogenetic distribution of vitamin biosynthetic genes at the genus level throughout the GIT regions. Fig. S13. The important indicators for regional heterogeneity identified by Random Forest model. Fig. S14. The detection of causal interactions between structure of GIT microbiota and vitamin biosynthesis pathway. Fig. S15. Vitamin synthesis capabilities of 31 genomes assigned to the genera Salmonella and Escherichia. Fig. S16. Variability in differences of vitamin biosynthesis explained by diets. Fig. S17. The detection of causal interactions between Bacteroides and Fibrobacter and cobalamin biosynthesis. Fig. S18. The important indicators for dietary shifts identified by Random Forest model. [file 40168_2022_1298_MOESM1_ESM.docx]

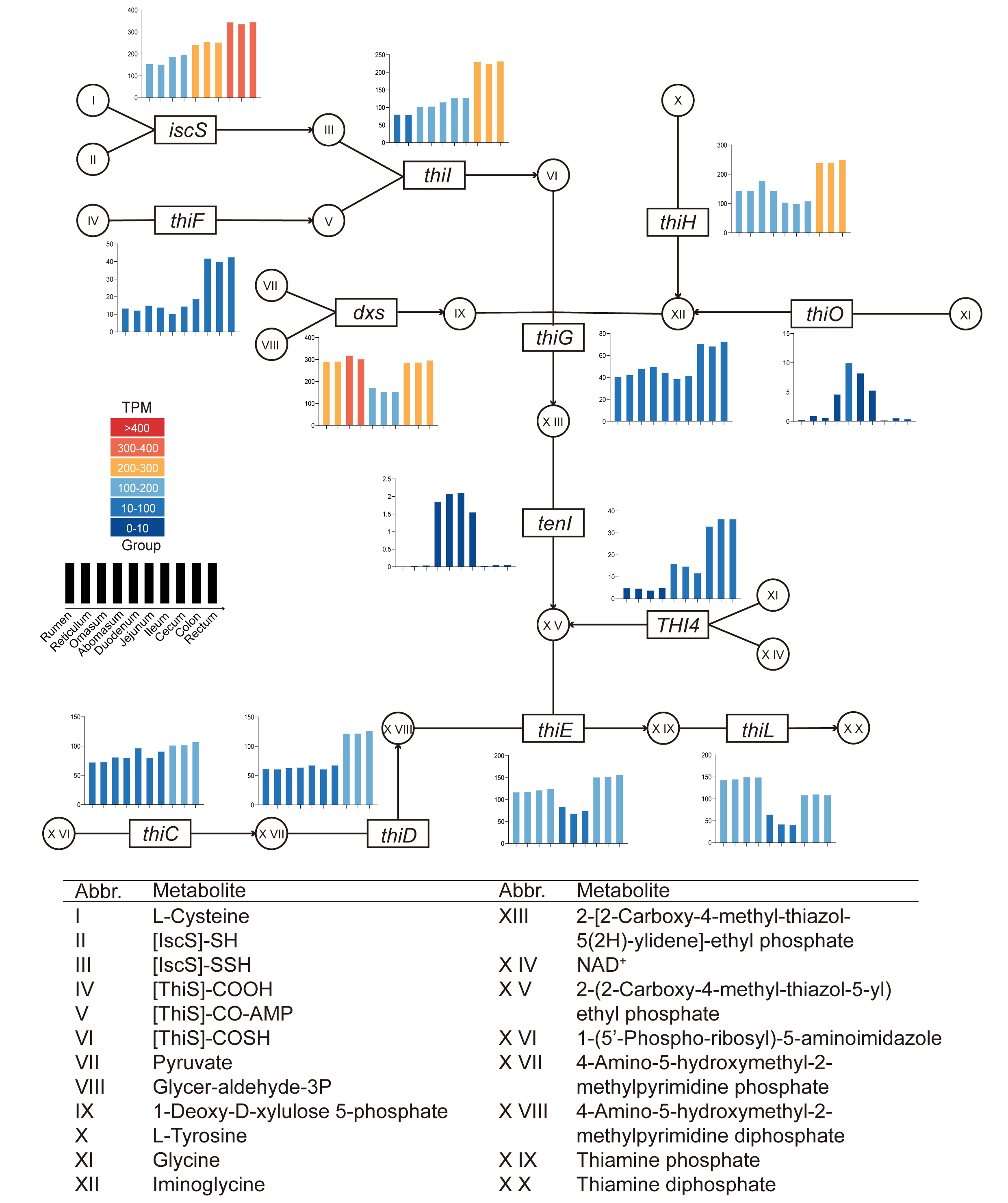


**Fig. S1 The biosynthesis pathway of thiamine.** The thiamine biosynthesis pathway contains 13 functional roles and 20 metabolites. Functional roles are represented by rectangles and metabolites are represented by circles. Each bar graph represents the relative abundance (TPM) of the corresponding functional role in each GIT region.


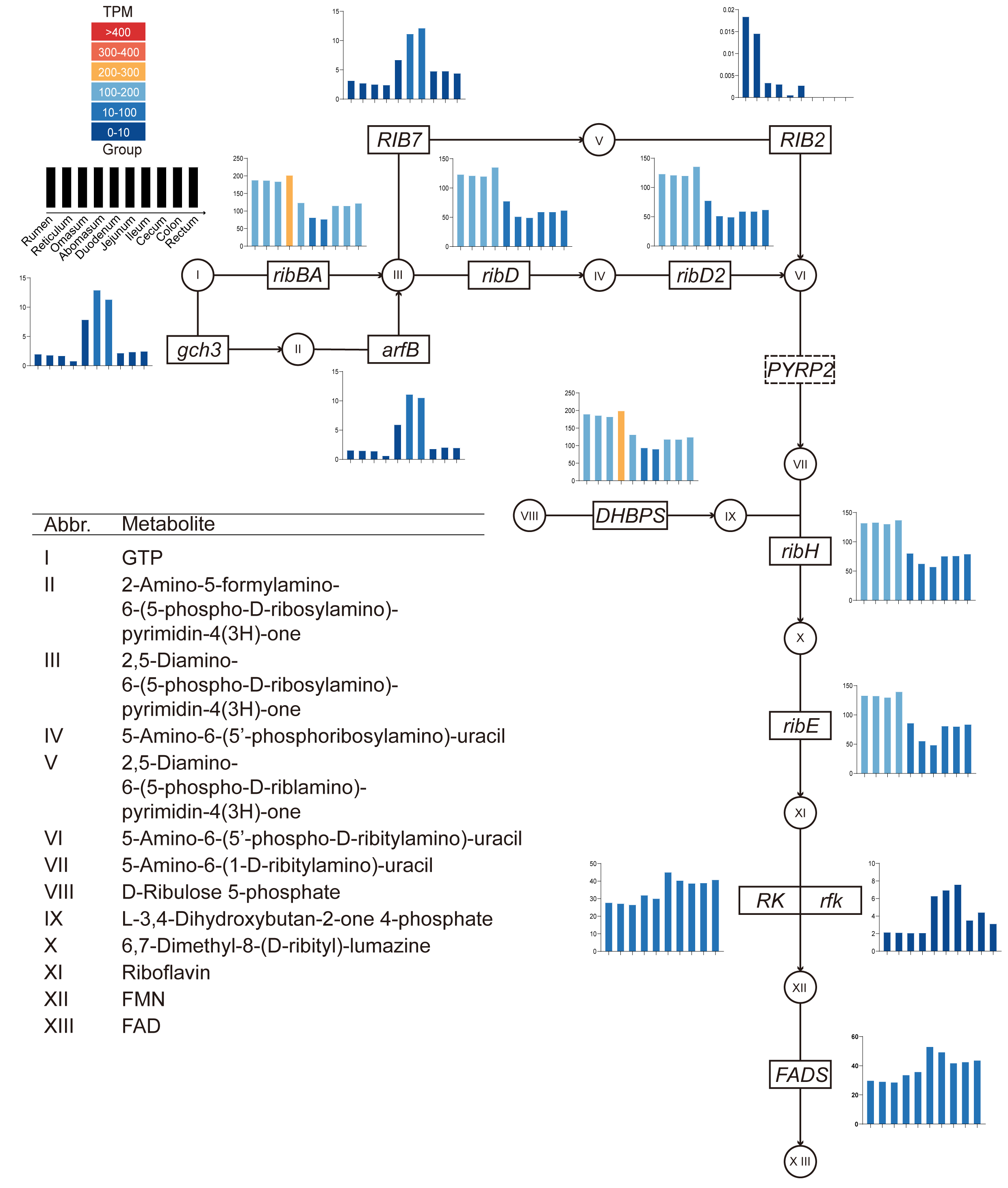


**Fig. S2 The biosynthesis pathway of riboflavin.** The riboflavin biosynthesis pathway contains 14 functional roles and 13 metabolites. The dotted rectangle represents the functional role missing in our prediction. Refer to **Fig. S1** for figure explanations.


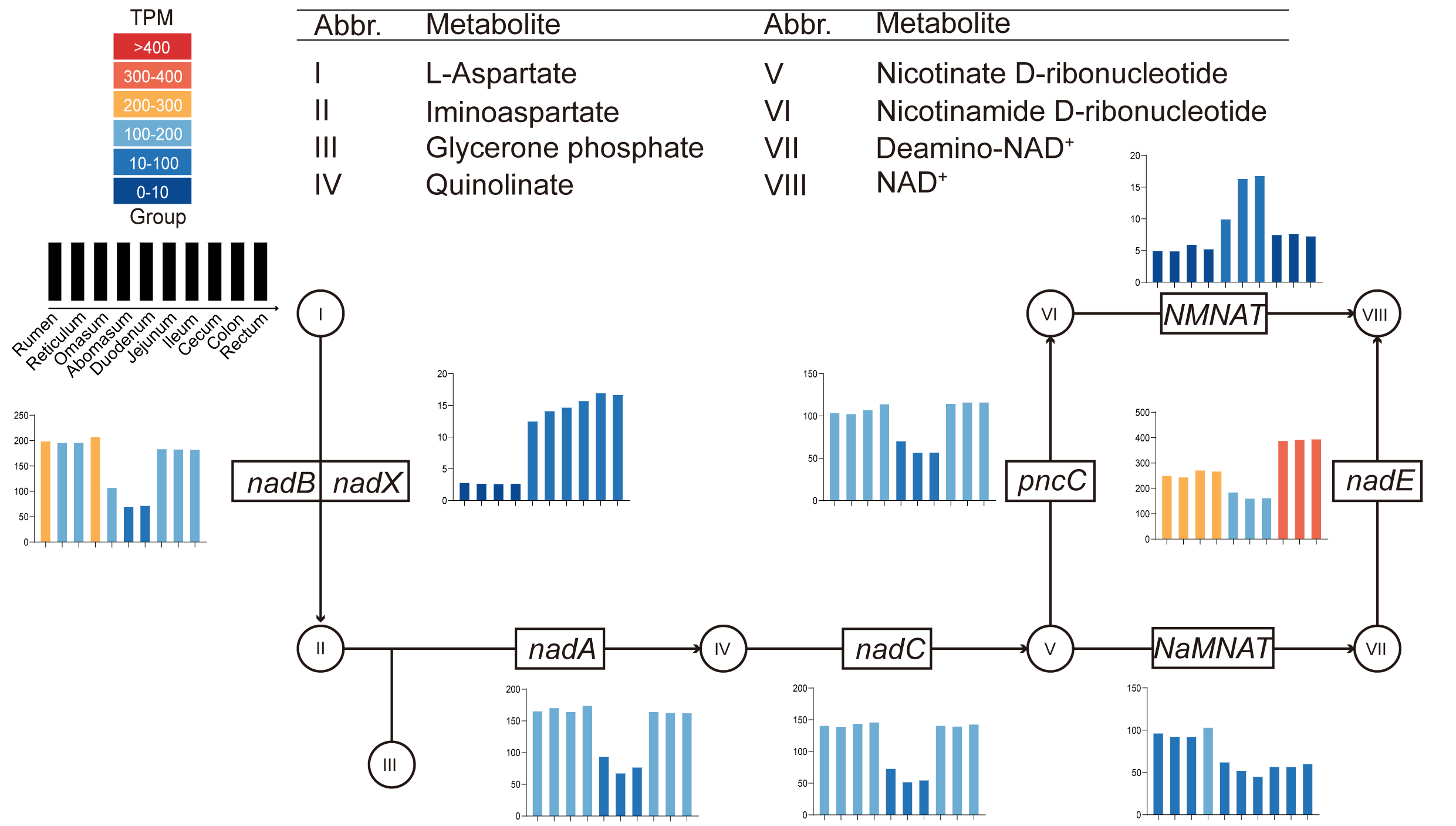


**Fig. S3 The biosynthesis pathway of niacin.** The niacin biosynthesis pathway contains 8 functional roles and 8 metabolites. Refer to **Fig. S1** for figure explanations.


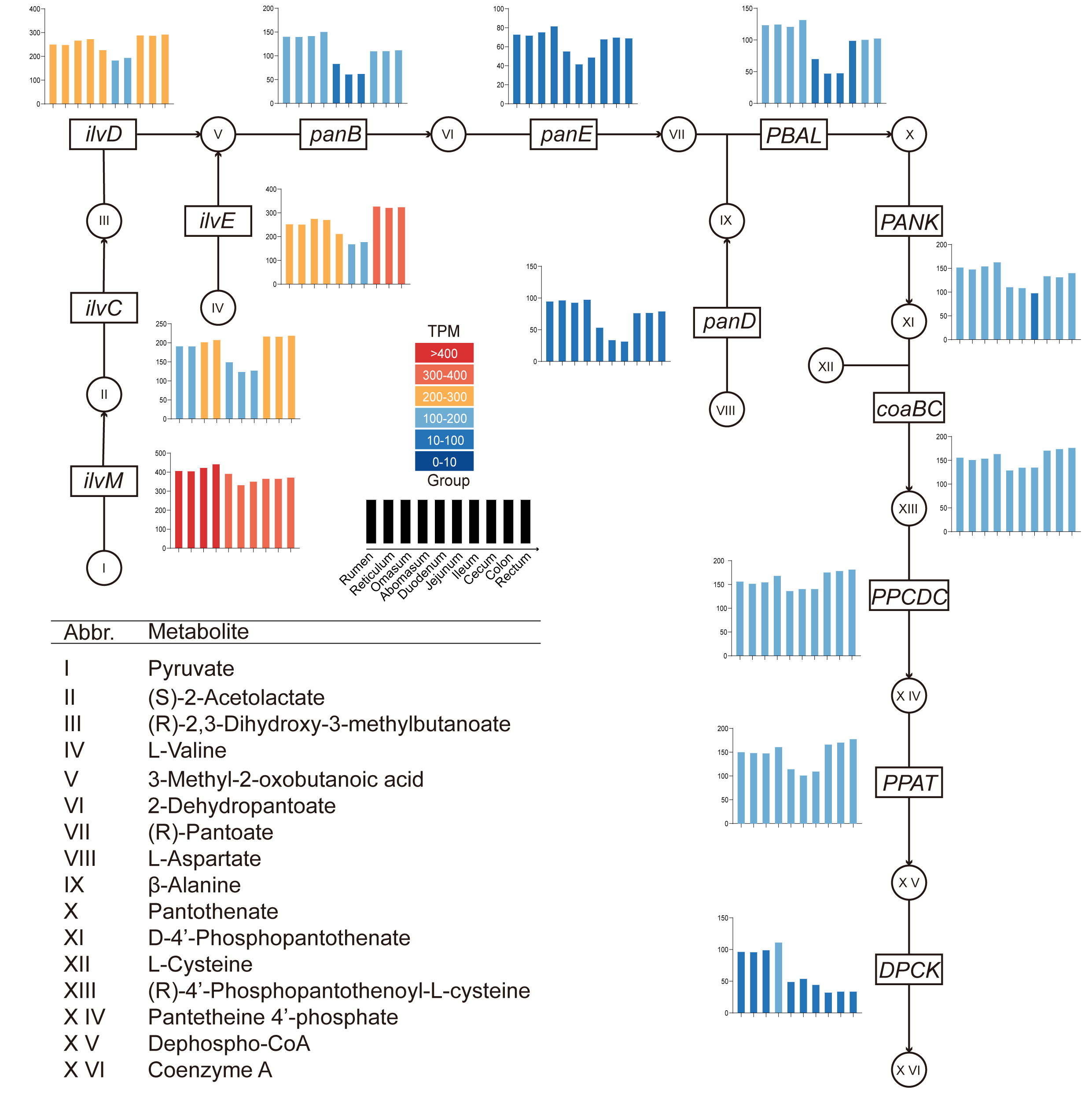


**Fig. S4 The biosynthesis pathway of pantothenate.** The pantothenate biosynthesis pathway contains 13 functional roles and 16 metabolites. Refer to **Fig. S1** for figure explanations.


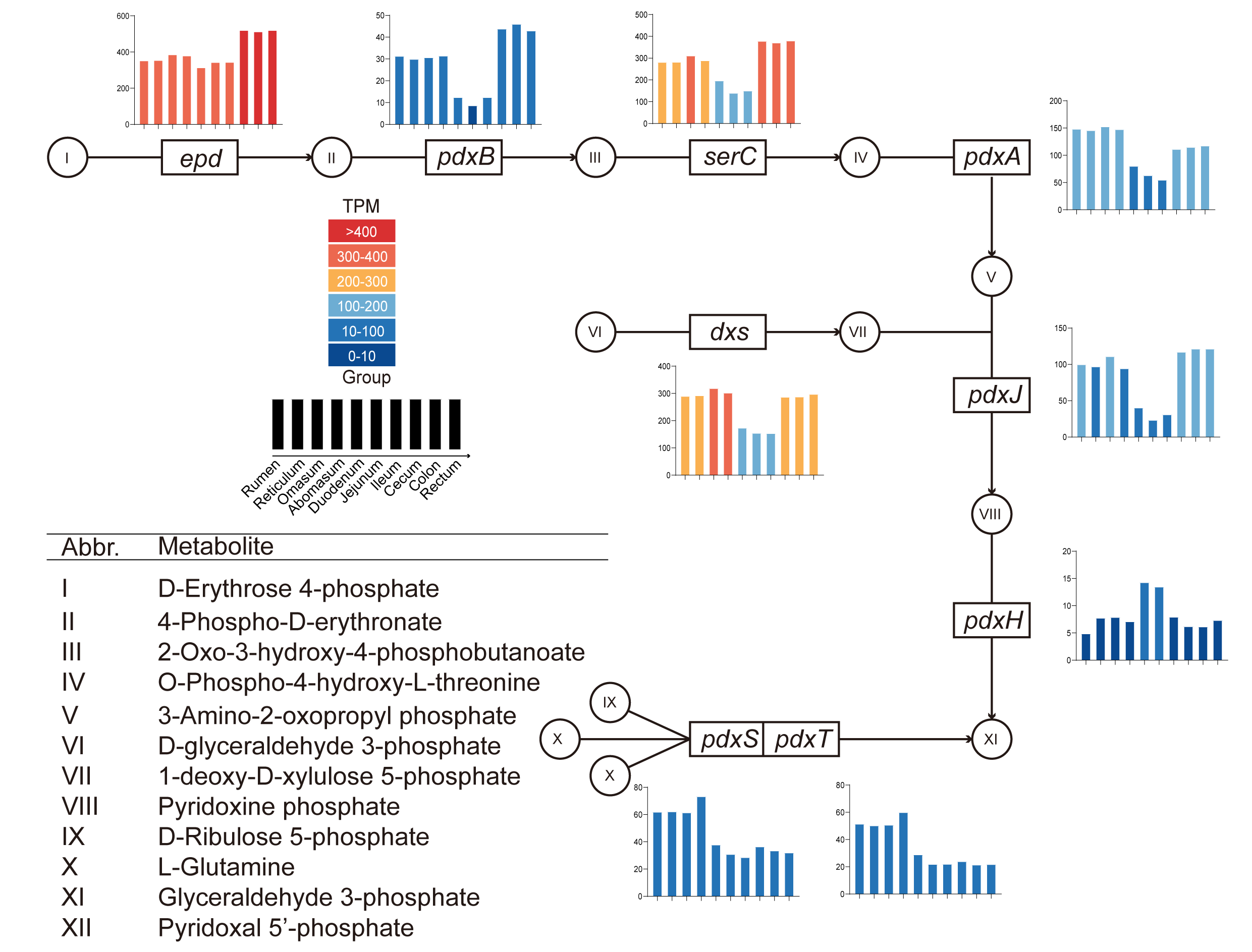


**Fig. S5 The biosynthesis pathway of pyridoxine.** The pyridoxine biosynthesis pathway contains 9 functional roles and 11 metabolites. Refer to **Fig. S1** for figure explanations.


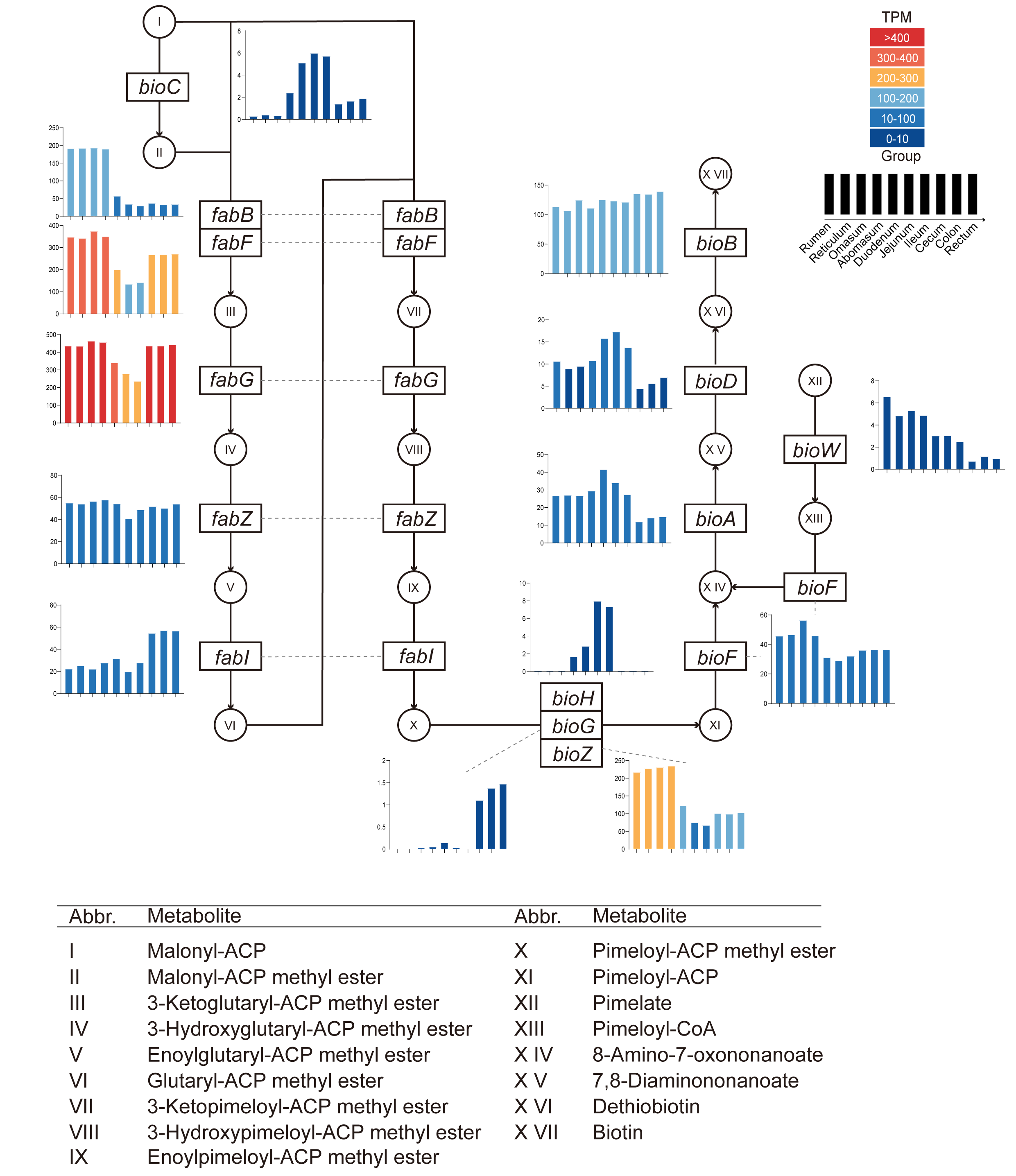


**Fig. S6 The biosynthesis pathway of biotin.** The biotin biosynthesis pathway contains 14 functional roles and 17 metabolites. Refer to **Fig. S1** for figure explanations.


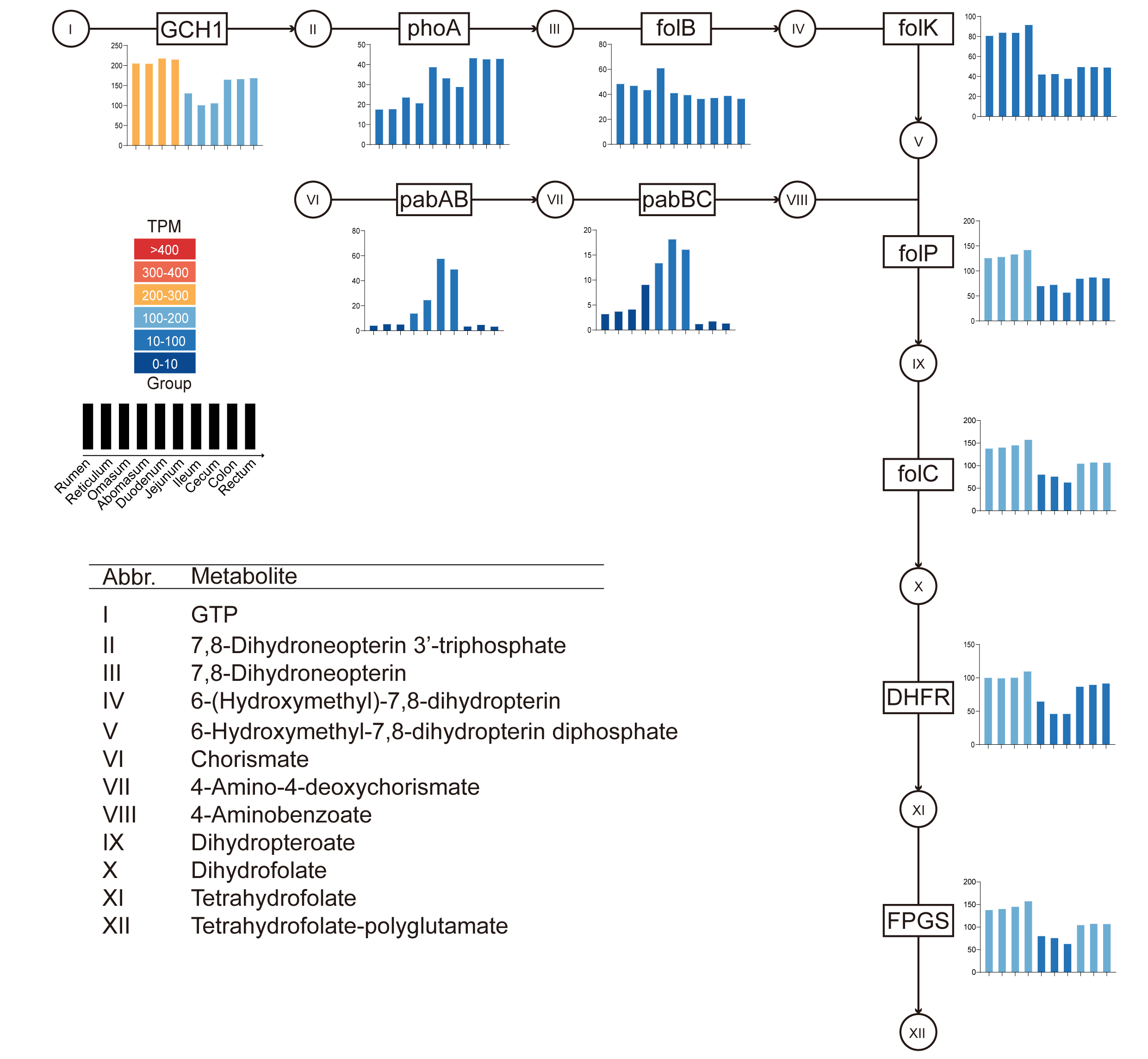


**Fig. S7 The biosynthesis pathway of folate.** The folate biosynthesis pathway contains 10 functional roles and 12 metabolites. Refer to **Fig. S1** for figure explanations.


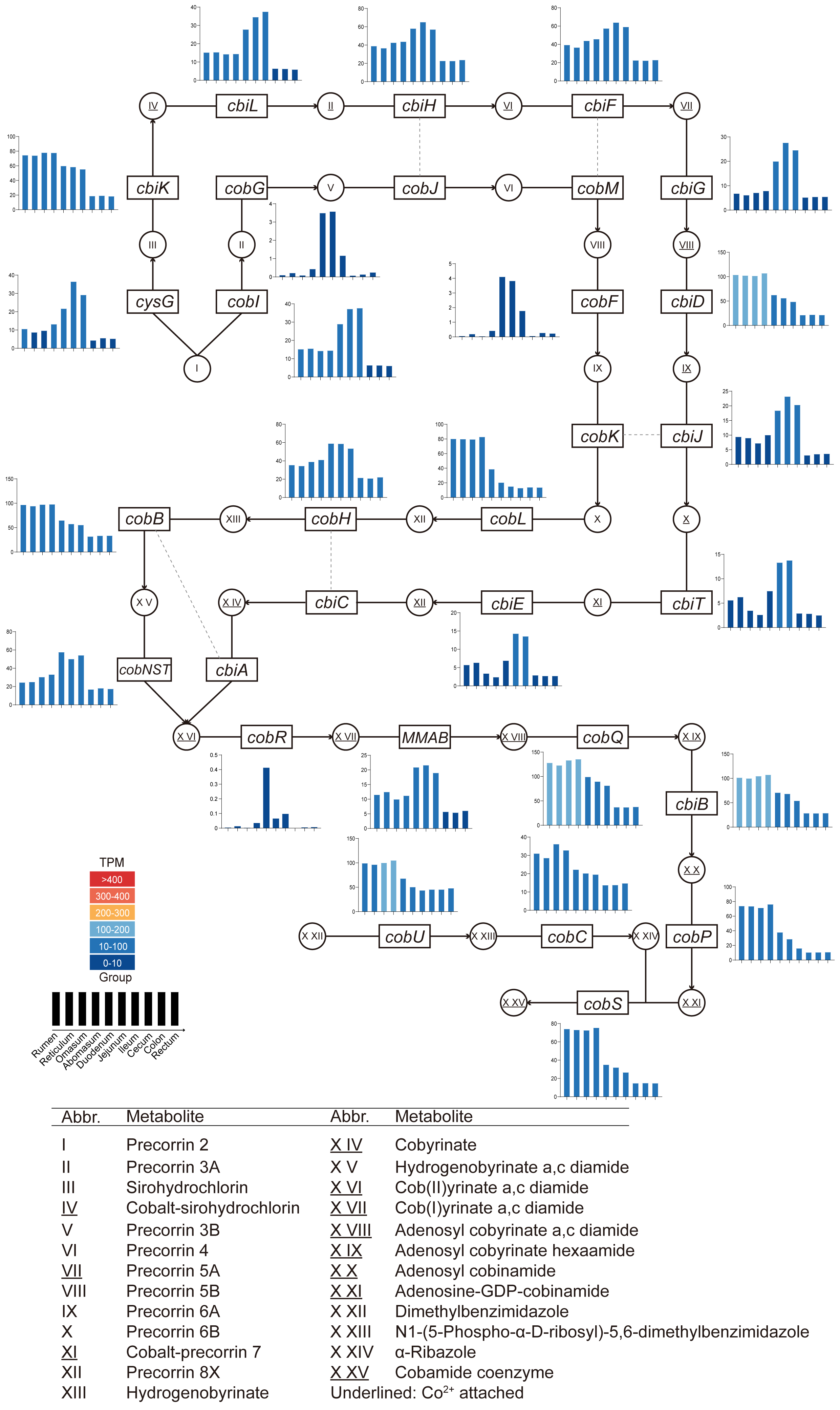


**Fig. S8 The biosynthesis pathway of cobalamin.** The cobalamin biosynthesis pathway contains 25 functional roles and 25 metabolites. Refer to **Fig. S1** for figure explanations.


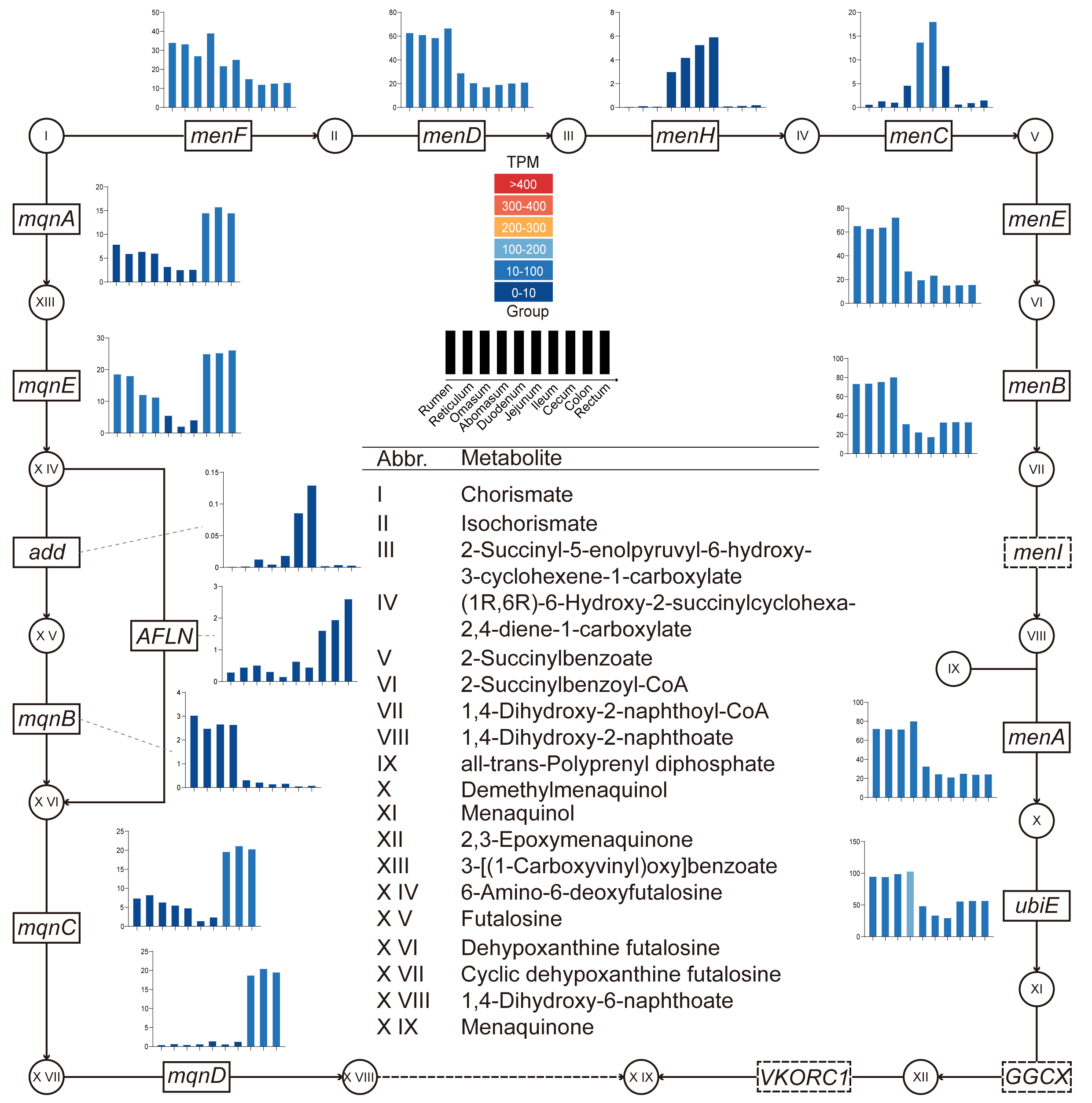


**Fig. S9 The biosynthesis pathway of menaquinone.** The menaquinone biosynthesis pathway contains 18 functional roles and 19 metabolites. The dotted rectangle represents the functional role missing in our prediction and the dotted line indicates that the biochemical reaction is unclear. Refer to **Fig. S1** for figure explanations.


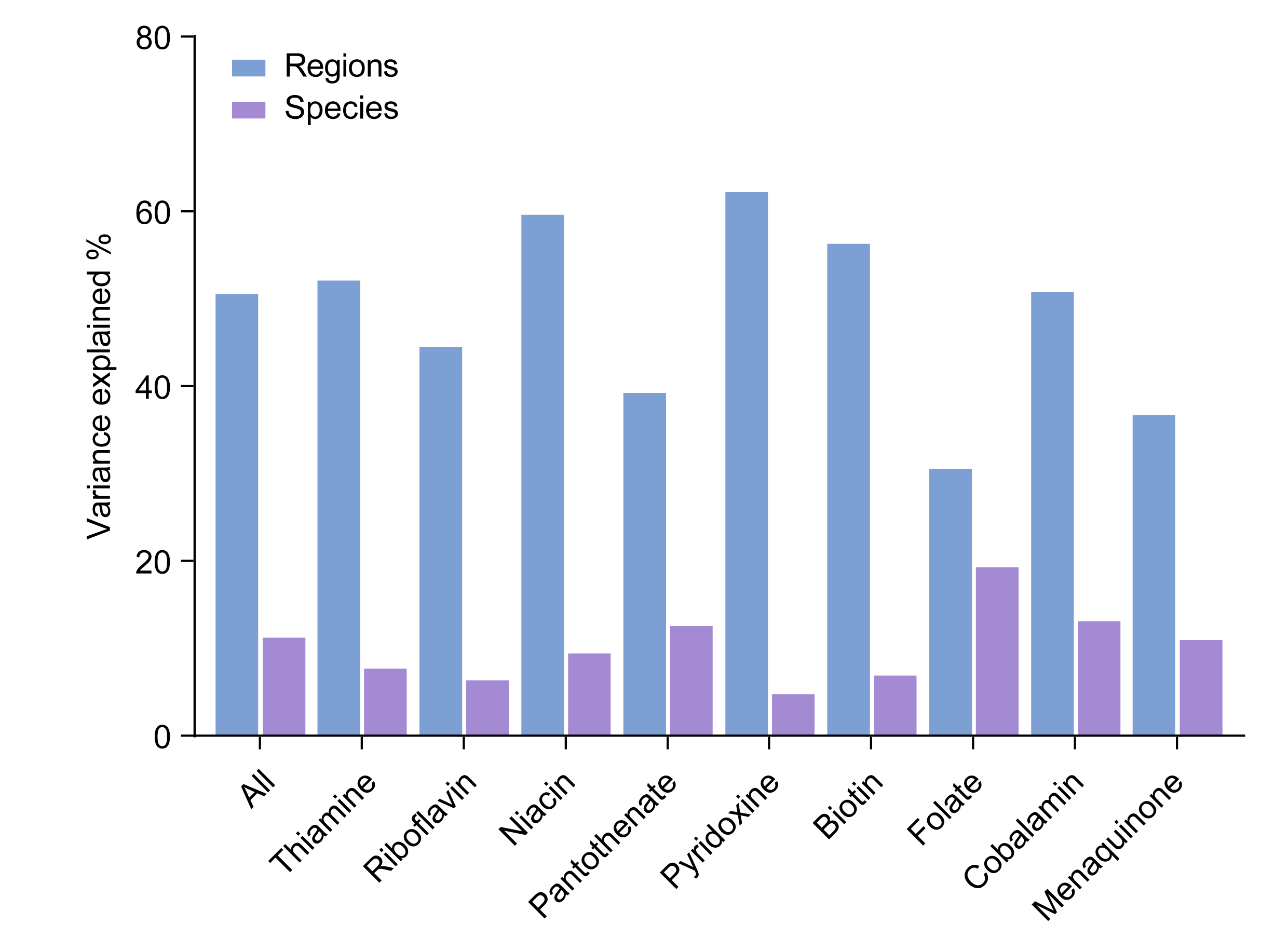


**Fig. S10** **Variability in differences of vitamin biosynthesis explained by regions and species.** The effects were assessed through variance partitioning analysis. “All” represents the dietary effect on all vitamin biosynthesis pathway abundances.


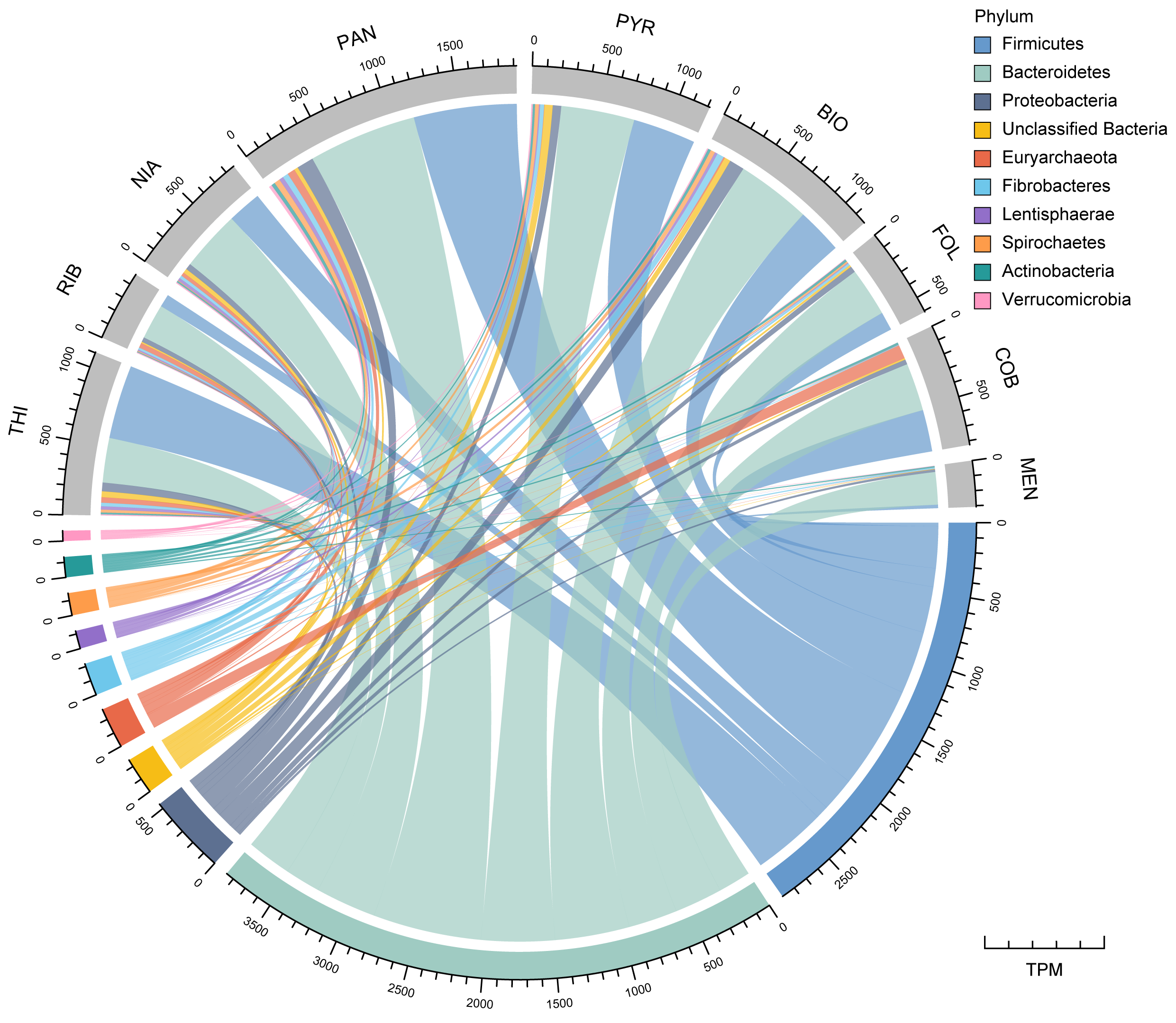


**Fig. S11 Chord plot of the distribution of vitamin biosynthetic genes among different phyla.** Each phylum is colored and each type of vitamin is shown in grey. Vitamin B and K_2_ are abbreviated as: thiamine (THI), riboflavin (RIB), niacin (NIA), pantothenate (PAN), pyridoxine (PYR), biotin (BIO), folate (FOL), cobalamin (COB), menaquinone (MEN).


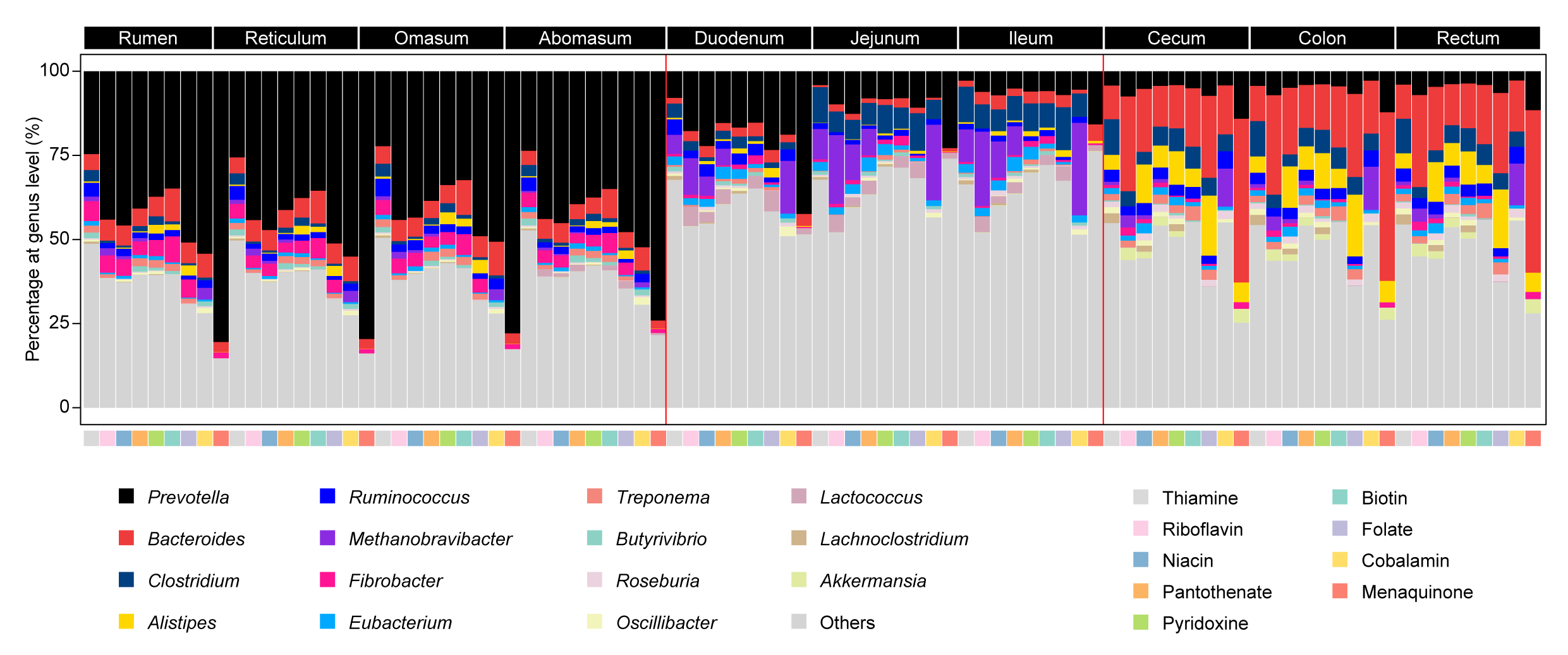


**Fig. S12 Phylogenetic distribution of vitamin biosynthetic genes at the genus level throughout the GIT regions.** Vitamin B and K_2_ biosynthesis-related microbial genes were assigned to NCBI-NR database for phylogenetic taxonomy at genus level.


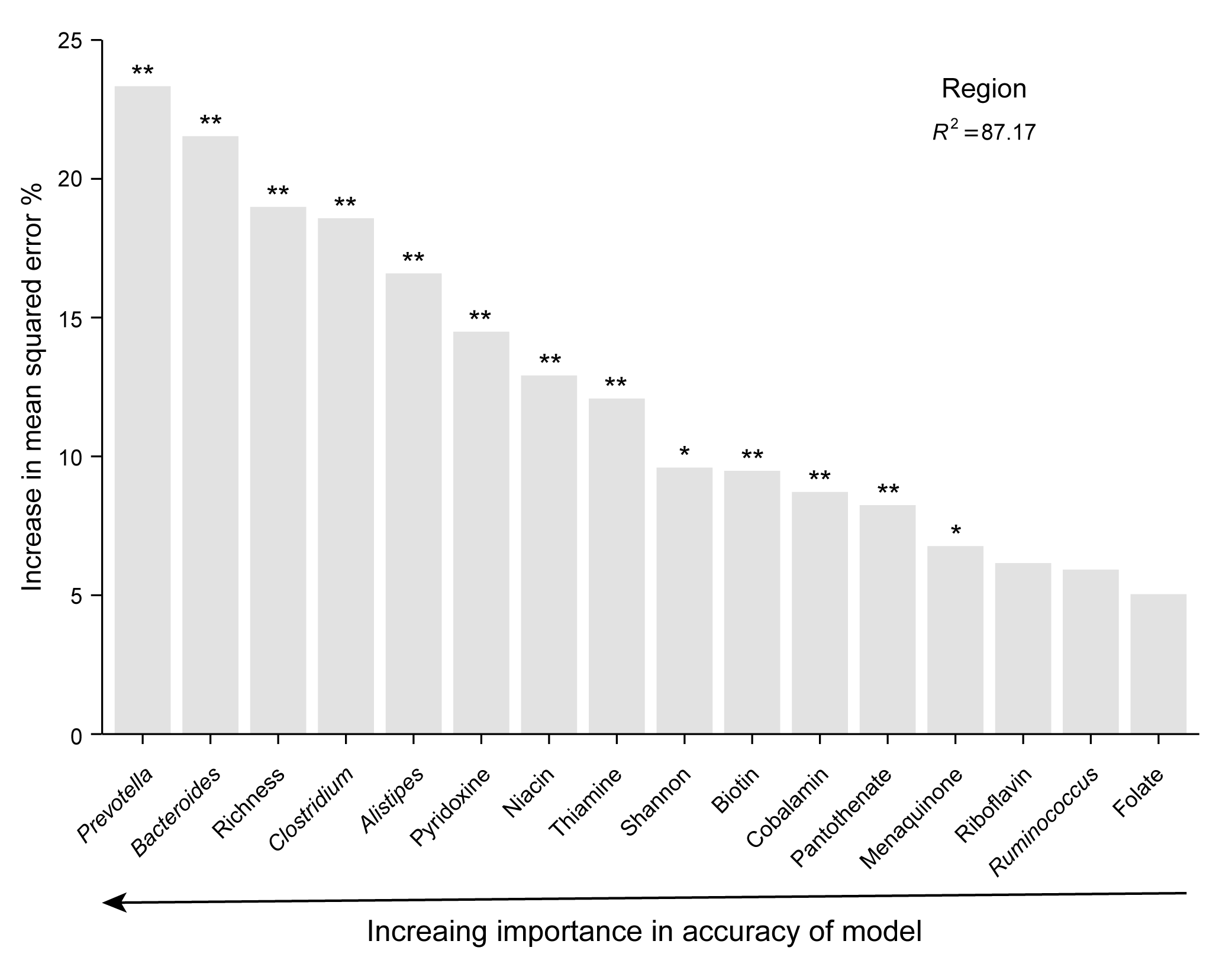


**Fig. S13 The important indicators for regional heterogeneity identified by Random Forest model.** The important indicators were identified by applying Random Forest regression of B and K_2_ vitamin biosynthesis pathway abundances, and abundances and alpha diversity of vitamin biosynthesis-related microbiota against GIT regions. Percentage increase in mean square error of variables were used to estimate the importance of these indicators, and higher mean square error % imply more important indicators. Significance levels are as follows: * *P* < 0.05 and ** *P* < 0.01.


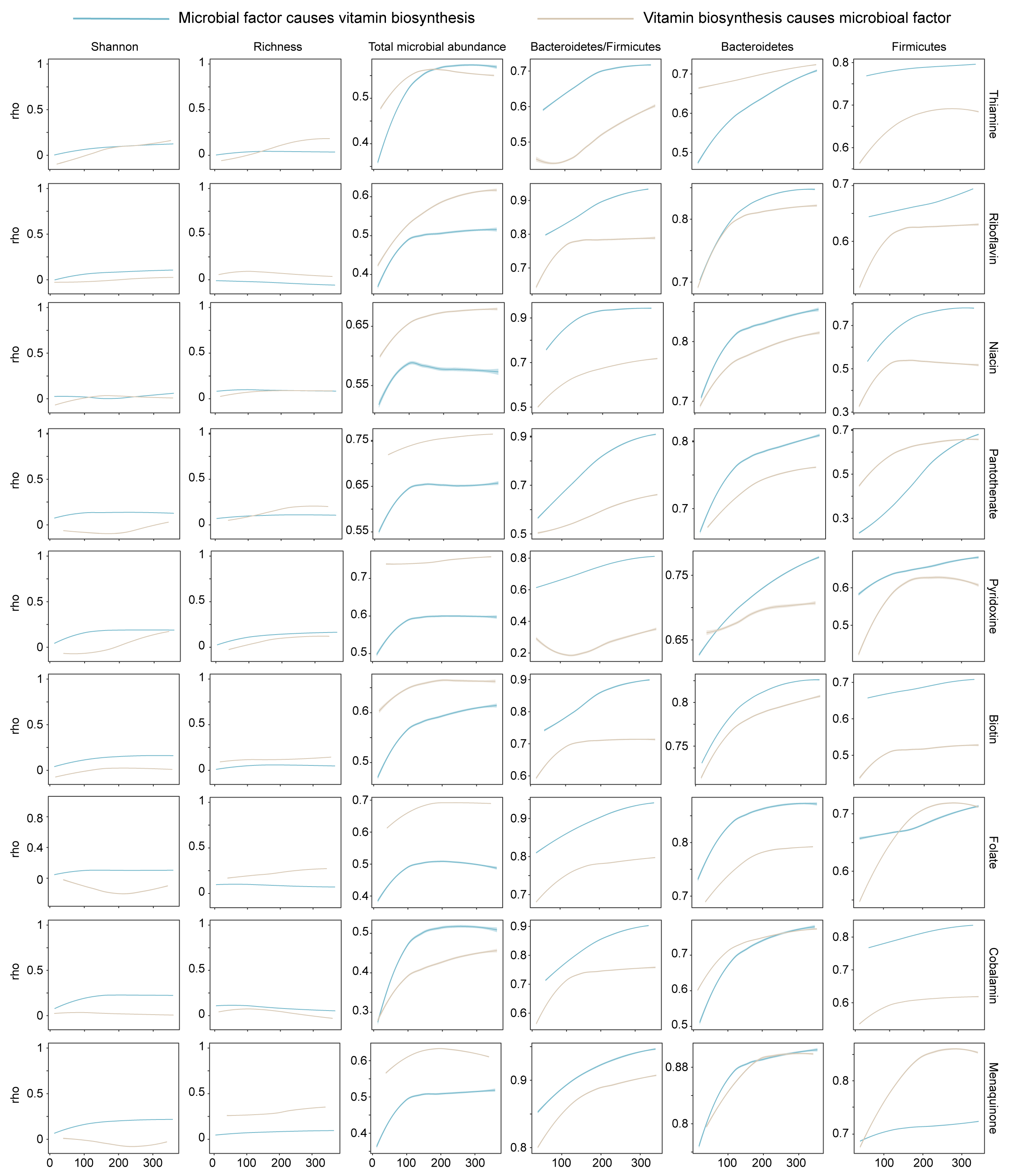


**Fig. S14 The detection of casual interactions between structure of GIT microbiota and vitamin biosynthesis pathway.** The casual interactions were detected using CCM model. The blue lines represent the strength of microbial factors forcing vitamin biosynthesis pathway abundances, and the brown lines represent the strength of vitamin biosynthesis pathway abundances forcing microbial factors.

**
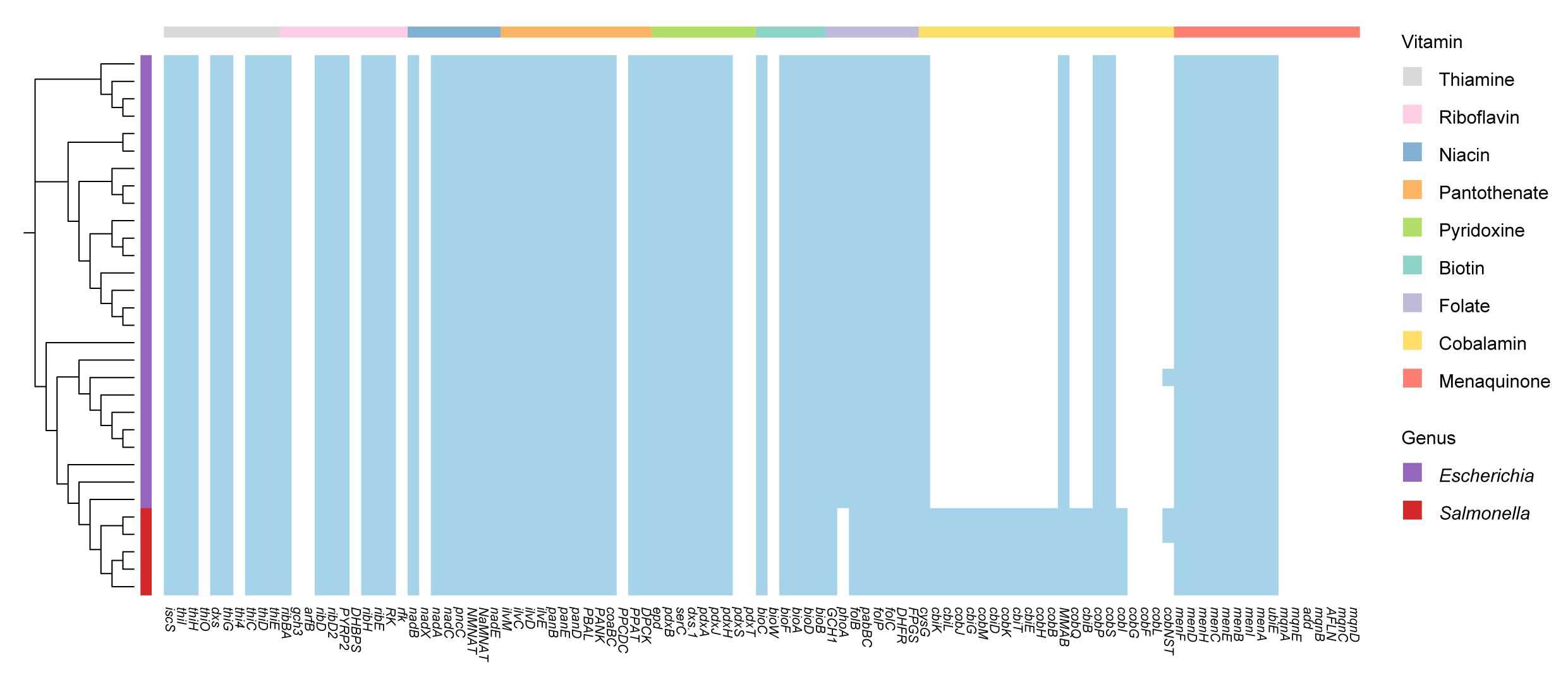
**

**Fig. S15 Vitamin synthesis capabilities of 31 genomes assigned to the genera *Salmonella* and *Escherichia*.** The maximum likelihood tree of the 31 genomes was constructed using PhyloPhlAn [53] and visualized using iTOL [55]. Heatmaps show the presence (lightblue) or absence (blank) of each essential functional role for vitamin *de novo* biosynthesis of 5 genomes assigned to *Salmonella* spp. and 26 genomes assigned to *Escherichia* spp..


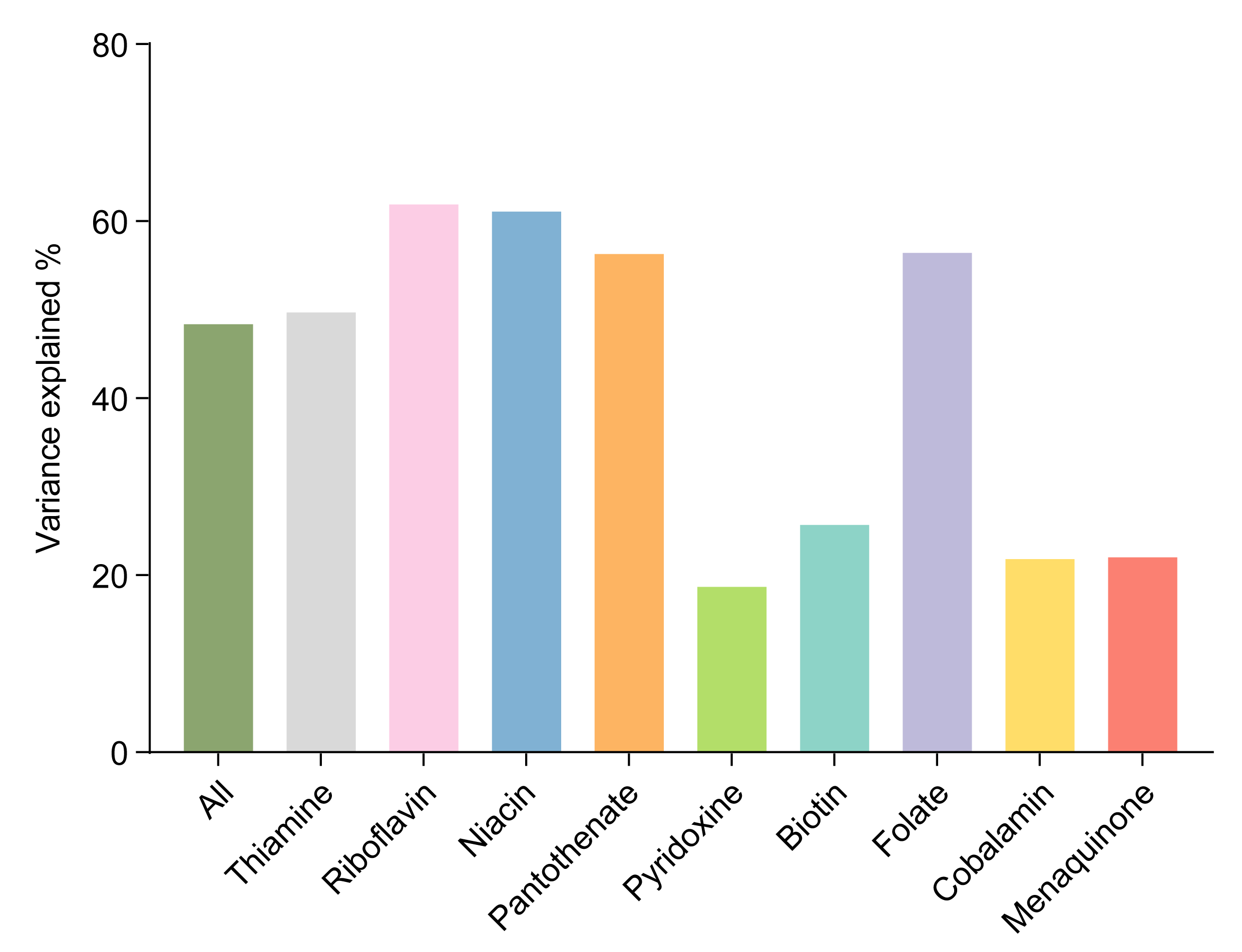


**Fig. S16** **Variability in differences of vitamin biosynthesis explained by diets.** The effects were assessed through variance partitioning analysis. “All” represents the dietary effect on all vitamin biosynthesis pathway abundances.


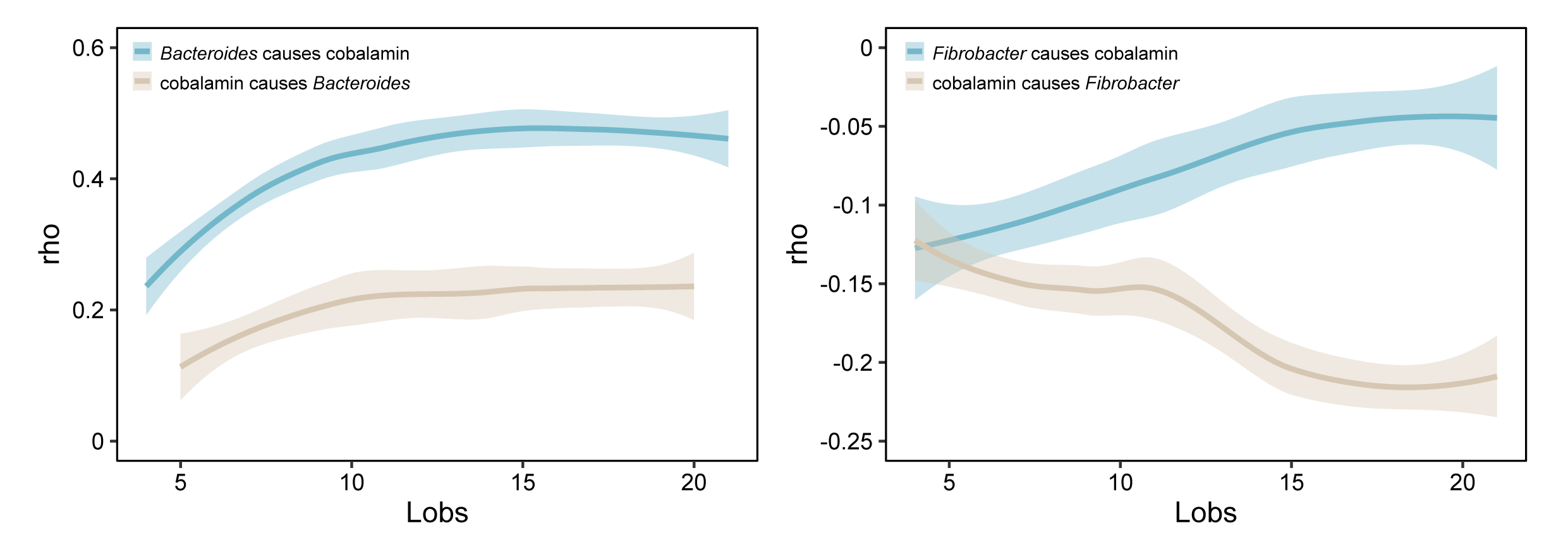


**Fig. S17 The detection of casual interactions between *Bacteroides* and *Fibrobacter* and cobalamin biosynthesis.** The causal interactions were detected using CCM model. The blue lines represent the strength of *Bacteroides* or *Fibrobacter* forcing cobalamin biosynthesis, and the brown lines represent the strength of cobalamin biosynthesis forcing the abundances of *Bacteroides* or *Fibrobacter*.


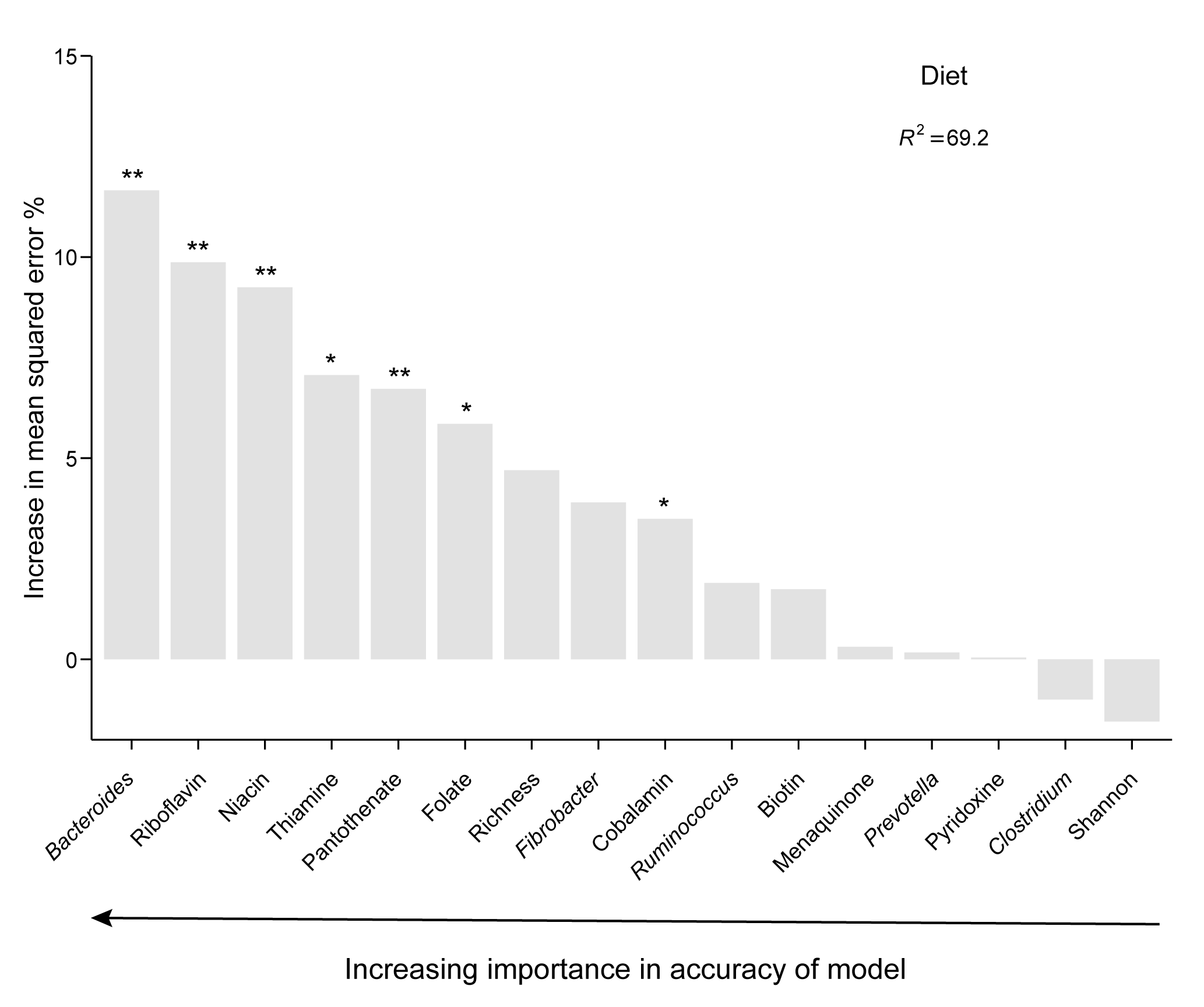


**Fig. S18 The important indicators for dietary shifts identified by Random Forest model.** The important indicators were identified by applying Random Forest regression of B and K_2_ vitamin biosynthesis pathway abundances, and abundances and alpha diversity of vitamin biosynthesis-related microbiota against diets. Percentage increase in mean square error of variables were used to estimate the importance of these indicators, and higher mean square error % imply more important indicators. Significance levels are as follows: * *P* < 0.05 and ** *P* < 0.01.
